# LEDGF/p75 Proteins with Alternative Chromatin Tethers Are Functional HIV-1 Cofactors

**DOI:** 10.1371/journal.ppat.1000522

**Published:** 2009-07-17

**Authors:** Anne M. Meehan, Dyana T. Saenz, James H. Morrison, Jose A. Garcia-Rivera, Mary Peretz, Manuel Llano, Eric M. Poeschla

**Affiliations:** 1 Department of Molecular Medicine, Mayo Clinic College of Medicine, Rochester, Minnesota, United States of America; 2 Biological Sciences Department, University of Texas, El Paso, Texas, United States of America; University of Geneva, Switzerland

## Abstract

LEDGF/p75 can tether over-expressed lentiviral integrase proteins to chromatin but how this underlies its integration cofactor role for these retroviruses is unclear. While a single integrase binding domain (IBD) binds integrase, a complex N-terminal domain ensemble (NDE) interacts with unknown chromatin ligands. Whether integration requires chromatin tethering *per se*, specific NDE-chromatin ligand interactions or other emergent properties of LEDGF/p75 has been elusive. Here we replaced the NDE with strongly divergent chromatin-binding modules. The chimeras rescued integrase tethering and HIV-1 integration in LEDGF/p75-deficient cells. Furthermore, chromatin ligands could reside inside or outside the nucleosome core, and could be protein or DNA. Remarkably, a short Kaposi's sarcoma virus peptide that binds the histone 2A/B dimer converted GFP-IBD from an integration blocker to an integration cofactor that rescues over two logs of infectivity. NDE mutants were corroborative. Chromatin tethering *per se* is a basic HIV-1 requirement and this rather than engagement of particular chromatin ligands is important for the LEDGF/p75 cofactor mechanism.

## Author Summary

Like other retroviruses, HIV-1 integrates a DNA copy of its genome into a host cell chromosome in each replication cycle. The resulting integrated proviruses are the basis for two important clinical problems: the inability to eradicate HIV-1 from the body and the permanent archiving of drug-resistant viruses. The DNA recombining steps catalyzed by the viral integrase are known, but the process as it occurs between the incoming virus and chromatin in cells is incompletely understood. LEDGF/p75 has been identified as an HIV-1 integration cofactor. If integrase is over-expressed in the absence of other viral components, it becomes linked to chromatin via LEDGF/p75. Whether this chromatin attachment function is the necessary and sufficient basis for its cofactor role in the viral life cycle has been unclear. The current work shows that HIV-1 cofactor function is preserved if the chromatin binding modules of LEDGF/p75 are replaced with widely disparate chromatin linkages. These results are evidence that chromatin tethering *per se* rather than connections to specific chromatin ligands is central to the LEDGF/p75 mechanism. They also have implications for targeting of lentiviral vectors within the human genome.

## Introduction

HIV-1 and other retroviruses use encoded integrase (IN) enzymes to catalyze permanent insertion of a cDNA copy of the viral genome into host DNA in each replication cycle [1]. Experiments with model DNA targets have defined the essential DNA-recombining steps. However, the process within the cell nucleus, where the viral pre-integration complex must negotiate nuclear transit, then access and insert into a chromosome, remains undefined in many respects [2]. Each of the two reaction participants is a complex macromolecular assembly and the chromatin fiber is also the site of numerous intricate processes, including transcription, DNA replication and diverse DNA repair activities, each of which involves formation of multi-protein machineries. Nuclear proteins have long been suspected to participate in retroviral integration [3] and recent studies have identified LEDGF/p75 as a lentivirus-specific replication cofactor [4]–[13]; see [14] and [15] for current reviews. LEDGF/p75 depletion impairs HIV-1 integration [7],[9],[12] and over-expression of the IBD produces substantial dominant-interfering activity [7],[8]. LEDGF/p75 has also been found to be a key determinant of the lentiviral bias for integration into transcription units [6],[12],[13]. Its normal cellular role appears to be modulating Pol II transcription, although this remains provisionally established, with the mechanism unknown [16].

Cofactor models currently conceive of LEDGF/p75 linking IN to unknown chromatin components. If lentiviral INs are over-expressed outside the viral context, they accumulate on chromatin [4], [5], [17]–[19] where they remain attached throughout the cell cycle [19]. Stable LEDGF/p75 knockdown shifts IN entirely to the cytoplasm [18]. LEDGF/p75 also protects IN from proteasomal degradation [20]. This latter stabilization effect is separable from chromatin tethering (it occurs in the cytoplasm with NLS-mutants or chromatin binding mutants of LEDGF/p75) and is insufficient for cofactor activity [7].

**Figure 1:**
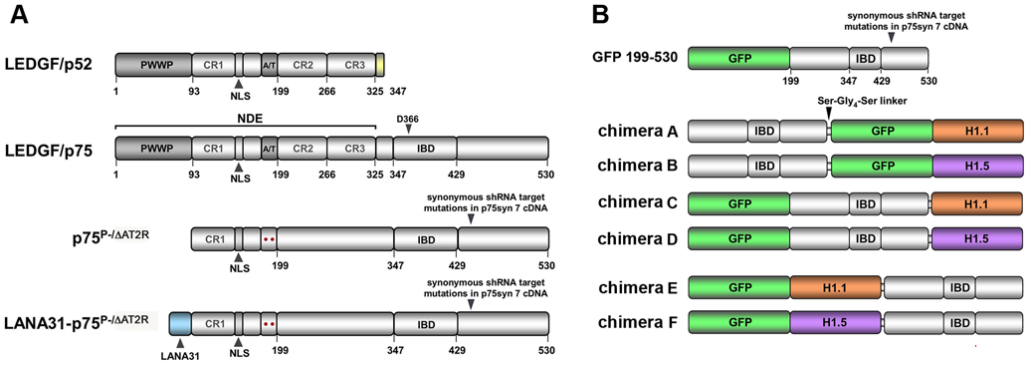
LEDGF/p75 and chimeric derivatives. Lengths of protein elements are drawn to scale. (A) Human LEDGF/p75 is shown with domains that have been identified. For LANA31-p75^P-/ΔAT2R^, the N-terminal 31 amino acids of the 1,162 amino acid KSHV LANA protein were fused in frame to p75^P-/ΔAT2R^. The latter consists of residues 94–530 of LEDGF/p75 with each A/T hook element also disabled by glycine substitution (red dot) of a functionally critical arginine [69]. The sequence of the LANA31 peptide is MAPPGMRLRSGRSTGAPLTRGSCRKRNRSPE. The first 23 residues (underlined) bind to the groove formed by adjacent molecules of core histone 2A and core histone 2B in the nucleosome core [29]. Note that all elements in the chimera retain native context polarity with respect to amino and carboxy termini. All constructs have 7 synonymous nucleotide changes at the RNAi target site. LEDGF/p52 (top) is the other splice variant of this gene. 333 amino acids in length, it has the same N-terminal 325 amino acids of LEDGF/p75 but lacks IN interaction, since a different 8 amino acid C-terminus (yellow segment) replaces residues 326–530 of LEDGF/p75. AT: A/T hook pair; CR1-3: charged regions 1–3; NLS: nuclear localization signal; • •: mutant AT hook; NDE: N-terminal domain ensemble involved in chromatin binding. (B) Linker histone chimeras. GFP-199-530 lacks the PWWP, AT hooks and NLS of LEDGF/p75; it does not bind chromatin [25] and is used as a control here. Linker histone (H1.1 and H1.5) chimeras A–F are illustrated. GFP–H1.1–199-530 and GFP–H1.5–199-530 (chimeras E and F) were eventually the most studied in viral replication experiments. A Ser-Gly-Gly-Gly-Gly-Ser (Ser-Gly4-Ser) linker was used as illustrated. All constructs have 7 synonymous nucleotide changes at the RNAi target site (illustrated for GFP-199-530). See Table 1 for a comparison of the properties of H1 and LANA31.

Interactions with chromatin and IN have been mapped to the LEDGF/p75 N- and C-terminal regions respectively (Figure 1A). The single, structurally discrete IN binding domain (IBD) is situated C-terminally [19],[21] and consists of four alpha helices [22]. Two inter-helical loops extend to interact with the IN core catalytic domain dimer interface [23] and one loop also interacts with the IN N-terminal domain [24]. In contrast to the IBD, the chromatin-binding end of the virologically functional tether is much less defined and its ligands are unknown. Chromatin attachment is mediated by a complex N-terminal domain ensemble (NDE), which occupies most of LEDGF/p75, up to residue 325 [25]. Minimally it includes a PWWP domain, the nuclear localization signal (NLS), a charged region 1 (CR1), and an A/T hook domain composed of two A/T hook elements [25],[26]. Deletion and domain transfer experiments suggest complex synergy of the various NDE elements, with the two most functionally critical being the PWWP domain and the A/T-Hook pair (Figure 1A). Deleting the PWWP domain disrupts LEDGF/p75 association with condensed chromosomes in mitosis [25]. Analyzed alternatively by biochemical analysis of sub-cellular fractions, PWWP domain deletion impairs high affinity chromatin association but additional deletion of the A/T Hook domain is required to abolish it [25]. The PWWP domain in conjunction with an adjacent region of 49 relatively charged amino acids (CR1) suffices to tether GFP to condensed chromosomes, but transfer of Triton-resistant binding to GFP is negligible without addition of the A/T hook pair [25]. Two additional relatively charged regions (CR2 and CR3) have no autonomous chromatin binding activity but appear to cooperatively enhance dominant effects of the other NDE elements, with full Triton-resistance achieved only when the donor to GFP extends from the N-terminus of LEDGF/p75 through CR3 [25]. Finally, the NLS region has also been reported to contribute to chromatin association [26].

Depletion of LEDGF/p75 clearly impairs the intra-nuclear progress of HIV-1 towards the integrated state, but the mechanism of the cofactor assistance the protein provides is unclear. Lacking explanation also is the evolutionary pressure driving strict conservation of LEDGF/p75-IN binding among all lentiviruses, yet only lentiviruses, despite substantial sequence variation in the IN dimer interfaces of the primate, feline, and ungulate lentivirus groups. A mutant deleted of the PWWP and A/T hook domains lost all chromatin association and was unable to rescue HIV-1 replication in LEDGF/p75-depleted cells [7], as did a similar mutant in murine knockout cells [12]. However, whether chromatin attachment *per se* is the necessary and sufficient mechanism *in vivo* is unknown. The question is compounded by limited understanding of the individual NDE elements and their natural roles [27],[28]. Our goal here was to test the chromatin tethering hypothesis explicitly. We sought also to discriminate between two models of LEDGF/p75 action. The first posits NDE-specific functional roles in enabling integration. The alternative scenario is one in which the primary role for LEDGF/p75 reflects its ability to meet two criteria: binding chromatin strongly and possessing a domain (the IBD) to which lentiviral IN proteins could evolve high-affinity binding without unsolvable constraints on their catalytic or other viral functions. If the latter is the case, it could signify a general requirement for which other retroviral genera have evolved alternative chromatin capture mechanisms, especially if their IN proteins are constrained on functional grounds from evolving dimer interfaces that bind the IBD. We replaced the LEDGF/p75 NDE with selected, divergent chromatin binding modules: two variants of the human linker histone H1 and a 31 amino acid peptide derived from the amino terminus of Kaposi's sarcoma herpes virus (KSHV) latency associated nuclear antigen (LANA) [29]. These elements were chosen for particular criteria. They are unrelated to any LEDGF/p75 domains and are well-characterized at the molecular level. H1 and the KSHV LANA peptide also differ strongly in evolutionary origin, natural role, size and structure. Their chromatin ligands contrast sharply as well: H1 binds outside the nucleosome, to DNA, while the LANA peptide binds inside the nucleosome core, to protein. We hypothesized that fusion of one or more of these chromatin-binding domains to C-terminal regions of LEDGF/p75 that contain the IBD could result in chimeric proteins that would rescue HIV-1 IN tethering and HIV-1 infection in CD4+ human T cells that are LEDGF/p75-deficient and/or express interfering IBD fragments. We additionally tested NDE-mutants of LEDGF/p75. Our results show that neither a specific chromatin ligand, nor even a basic location or molecular class of ligand within the chromatin fiber is required.

## Results

### H1 and LANA31 confer high-stringency chromatin binding to LEDGF/p75 C-terminal segments

Histones are of two general types. The four core histones (H2A, H2B, H3 and H4) form a protein octet at the center of each 10 nM diameter nucleosome, the basic unit of chromatin. In contrast, the linker histone H1 binds externally, to the stretch of DNA entering and exiting each nucleosome [30],[31]. In the absence of H1, the nucleosome consists of 147 base pairs of DNA wrapped around the octet in 1.65 super-helical turns, while in its presence the resulting “chromatosome” contains two complete turns, with 168 bp of DNA [32]–[34]. The bending and fastening of DNA fosters higher-order coilings e.g., the 30 nm fiber comprised of 6–8 nucleosomes. We prioritized H1 as a chromatin-binding module for several reasons. Mammalian eukaryotic chromatin contains approximately 0.5–1.0 histone H1 molecules per nucleosome, with a 1∶1 stoichiometry reported for lymphocytes [35]–[37]. This chromatin-associated protein is thus ubiquitous yet situated external to the nucleosome core [36], [38]–[41]. In addition, GFP-H1 fusions are known to retain normal chromatin interaction [33],[39] and cells are broadly tolerant of manipulations or even knockouts of individual H1 variants [31]. Finally, H1 histones exchange among chromatin binding sites in both condensed and uncondensed chromatin [38],[39]. We predicted this would allow introduced chimeras to achieve dynamic equilibrium with endogenous H1 molecules. Six human H1 variants with varying chromatin mobility exist [42]. Of these, we selected H1.1 and H1.5 because they represent the ends of this mobility spectrum with H1.1 having the fastest off rate and H1.5 the slowest [33]. Six H1 chimeras (chimeras A–F, Figure 1B) were made by placing amino acids 199–530 of LEDGF/p75 within three different in-frame permutations with GFP and either human H1.1 or H1.5. In chimeras E and F, GFP-tagged H1.1 and H1.5 are situated N-terminally, mimicking the location of the LEDGF/p75 chromatin binding domain ensemble. To allow expression in cell lines with potent stable RNAi against LEDGF/p75, all constructs have 7 synonymous mutations in the shRNA target sequence [7],[19].

**Figure 2:**
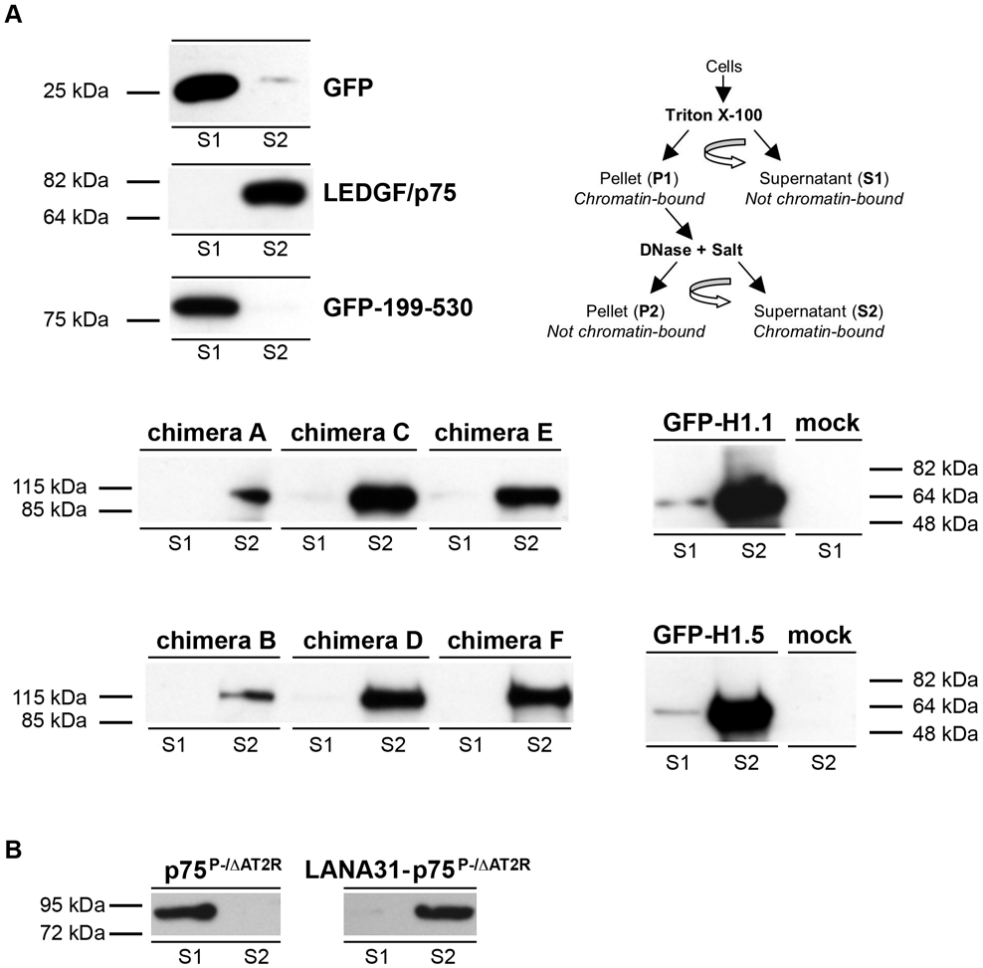
Chromatin binding assays. (A) H1 chimeras are stringently chromatin bound. The sub-cellular fractionation scheme is summarized in the diagram. See Materials and Methods for details and [25] for complete assay validation with varied control proteins. Here the S1 and S2 fractions are shown. The H1 chimeras A–F and GFP-H1.1 and GFP-H1.5 were detected with anti-GFP. Chimeras A–F had predicted molecular mass (∼93 kDa) as did GFP-H1.1 and GFP-H1.5 (∼57 kDa). GFP199-530 displays no chromatin binding and like GFP is found in the S1 fraction. LEDGF/p75 is tightly chromatin bound and fractionates solely to S2. (B) Addition of the LANA31 peptide to the N-terminus of p75^P-/ΔAT2R^ converts it to a chromatin binding protein found in S2.

Chimeras A–F and several control proteins were analyzed by immunoblotting, biochemical fractionation, and confocal imaging. Sub-cellular fractionation following a well-characterized protocol [7],[25] revealed that all six H1.1 or H1.5 chimeras are of predicted size and are found in the strongly chromatin-bound (S2) fraction, which is Triton-resistant and requires DNAse and salt treatment to mobilize (Figure 2A). Thus, the proteins display high-stringency chromatin association similar to LEDGF/p75. Control analyses showed this required the H1 moiety (Figure 2A, see GFP-199-530). While such biochemical assays average the properties of cells in all cell cycle phases, confocal imaging confirmed each chimera to be nuclear and documented association with both uncondensed and condensed mitotic chromatin (Figure S1A). In this respect they behaved identically to GFP-H1.1 and GFP-H1.5, while proteins lacking the linker histone, such as GFP-199-530, were un-associated with chromosomes.

LANA31, the 31 N-terminal amino acids of the KSHV LANA protein [29], was then used to establish an informative contrasting linkage with chromatin. Table 1 summarizes comparisons of this peptide with histone H1. KSHV is a gamma-herpes virus that achieves latency by persisting as a DNA episome [43]. LANA links repeats in the centromere-lacking episome to chromatin, enabling it to partition with the host cell genome at mitosis [44]. However, only the 23 N-terminal residues of the 1,162 amino acid KSHV LANA protein establish the chromatin linkage, via a tight hairpin structure formed by residues 5–13 that inserts with high specificity into a groove formed between core histones 2A and 2B [29]. Thus, in contrast to H1, this peptide binds inside the nucleosome core, and to a protein rather than nucleic acid ligand. We fused the LANA31 peptide in frame to the N-terminus of p75^P-/ΔAT2R^ (Figure 1), a LEDGF/p75 mutant that retains the LEDGF/p75 NLS and is nuclear, but lacks detectable chromatin association because the PWWP domain is deleted and each A/T hook is disabled by glycine substitution of a critical arginine residue [25]. As shown in Figure 2B, p75^P-/ΔAT2R^ segregated in the non-chromatin bound (S1) fraction whereas the addition of LANA31 caused strict, S2-fractionating chromatin association. This was cell cycle-resilient, as immunofluorescence analyses confirmed condensed and uncondensed chromatin association was conferred by the peptide (Figure S1B).

**Table 1:** Comparison of Substitute Chromatin Binding Modules.

Linker Histone (human H1.1 and H1.5)	LANA31 peptide
• Intra-chromatin ligand is DNA [36],[41].	• Intra-chromatin ligand is protein [29].
• Binds outside nucleosome [38].	• Binds inside nucleosome [29].
• Primary functions are diverse, include regulating nucleosome architecture [36].	• Primary function is chromatin-tethering of a large DNA virus genome [29],[44].
• Size approximately equal to p75 NDE [36].	• About 10 times smaller than p75 NDE [29].
• Well-characterized, known to maintain phenotype as GFP fusion [38].	• Well-characterized, known to tether GFP to chromatin [29].
• Variants with different chromatin on-off rates (H1.1 fast, H1.5 slow) [33].	• No variants [29].

### Chimeras tether IN to chromatin

**Figure 3:**
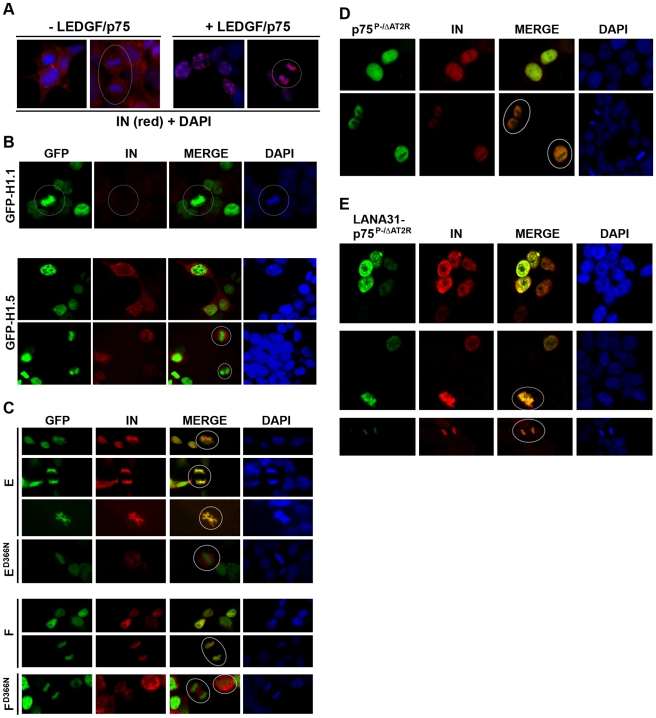
IN tethering by H1 and LANA31 chimeras. (A) Intracellular location of HIV-1 IN. Confocal micrographs of LH4 cells are shown. These cells stably express HIV-1 IN (red) and are also stably depleted of LEDGF/p75 by shRNA expression. When LEDGF/p75 is re-expressed in LH4 cells with an shRNA-resistant cDNA (right), HIV-1 IN re-localizes to the nucleus as a chromatin-tethered protein. Data not shown reveals two additional aspects: (i) re-expressed LEDGF/p75 co-localizes tightly with IN; (ii) detector gain is higher (∼1000) in the absence of LEDGF/p75 on left, compared with ∼600 in the (+) LEDGF/p75 images at right because IN is much more abundant. Some mitotic cells are pointed out with circles. (B) GFP-H1.1 (top panel row) proteins expressed in LH4 cells are chromatin-bound but do not interact with HIV-1 IN. Mitotic cells are circled. The stably expressed IN is poorly visualized because it is not protected as in C–E. For this reason, the lower two panel rows show GFP-H1.5 with additional IN co-transfected to facilitate imaging. (C) IN tethering. H1 chimeras E and F tether IN to chromatin but E^D366N^ and F^D366N^ do not. Note again that additional IN was transiently cotransfected with the D366N mutants to facilitate imaging. Mitotic cells are circled. Chimeras A, B, C and D produced the same results in about 90% of cells, although in contrast to chimeras E and F, slight non-overlap of GFP and IN signal was detected in occasional cells (data not shown). (D,E) p75^P-/ΔAT2R^ binds HIV-1 IN and traps it in the nucleus, but does not tether it to chromatin. In contrast the LANA31-p75^P-/ΔAT2R^ tethers. Note discrete foci of LANA31-p75^P-/ΔAT2R^ in interphase nuclei in E. Mitotic cells are circled.

To be capable of reconstituting LEDGF/p75 function, the H1 and LANA31 chimeras should at a minimum rescue chromatin-tethering of over-expressed HIV-1 IN. The H1 chimeras did so (Figure 3C), producing confocal imaging patterns similar to full-length LEDGF/p75 (Figure 3A). H1 chimeras E (GFP-H1.1-199-530) and F (GFP-H1.5-199-530) consistently displayed the strictest phenotype in repeated experiments, with no un-tethered IN remnant, and are compositionally the most straightforward. Therefore they were utilized for subsequent work. In contrast to the H1 chimeras, GFP-H1.1 and GFP-H1.5 bound chromatin but failed to relocate IN, indicating that the IBD linkage is necessary (Figure 3B). Mutation of LEDGF/p75 Asp366 to Asn (D366N) is known to specifically disrupt interaction of the IBD with the HIV-1 IN dimer by removing a critical negative charge involved in polar interactions with the connector peptide linking IN alpha helices 4 and 5 [23]. When introduced into the chimeric histone proteins, this single amino acid change left their chromatin-binding properties unchanged but resulted in failure to re-localize IN, confirming specificity further (Figure 3C).

LANA31-p75^P-/ΔAT2R^ also tethered IN to chromatin, again throughout the cell cycle (Figure 3E). However, LANA31-p75^P-/ΔAT2R^ and its associated IN displayed a different chromatin association pattern than H1.1- or H1.5-tethered IN, producing a more variegated, punctate appearance (Figure 3E). In contrast, the parental p75^P-/ΔAT2R^ protein displayed a homogeneously diffuse nuclear pattern (compare Figures 3D & 3E). Note also that IN is still nuclear-trapped by the latter chimera (which retains the LEDGF/p75 NLS) but is not chromatin-trapped and remains un-tethered without the LANA31 peptide (Figure 3D).

**Figure 4:**
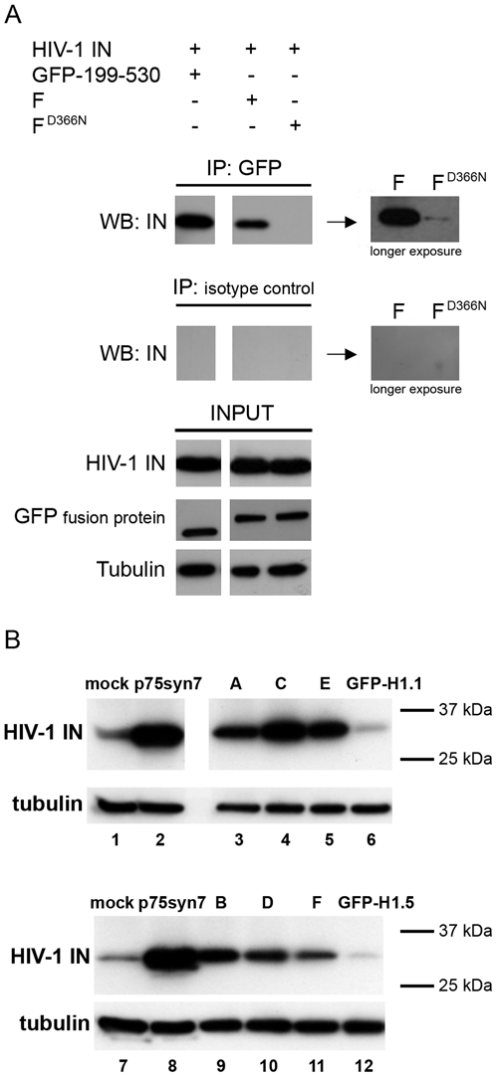
Chimeric proteins co-imunoprecipitate with HIV-1 IN and protect it from proteasomal degradation. (A) Co-immunoprecipitation with Myc-tagged HIV-1 IN. Proteins were immunoprecipitated with anti-GFP and immunoblotting was performed with anti-Myc. The positive control GFP199-530 pulls down HIV-1 integrase, as does H1 chimera F (GFP-H1.5-199-530). Prolonged over-exposure yielded a faint detectable IN band in the F^D366N^ lane (panel to right), with less than 1% of the F protein band intensity, suggesting slight residual interaction between IN and F^D366N^. (B) Chimeras protect HIV-1 IN in an IN stability rescue assay in LH4 cells. These cells are 293T cells that *(i)* are knocked down for endogenous LEDGF/p75 and *(ii)* contain a stably integrated HIV-1 IN expression plasmid [20]. In the absence of LEDGF/p75, only low IN levels are detectable (lanes 1 and 7). IN is protected by re-expression of LEDGF/p75 (p75syn7, lanes 2 and 8) or proteins that contain the LEDGF/p75 IBD (H1 chimeras A–E in lanes 3–5 and 9–11). In contrast, neither GFP-H1.1 or GFP-H1.5 protect (lanes 6 and 12).

The specific interaction of the histone constructs with HIV-1 IN was further confirmed by co-immunoprecipitation (Figure 4A). Note that the GFP-H1.5-199-530^D366N^ mutant also consistently pulled down HIV-1 IN, albeit much less than GFP-H1.5-199-530. We speculate that one or more additional mutations, e.g., F406A, may be needed to eradicate all detectable interaction of these proteins with IN [22]. We showed previously that LEDGF/p75 protects lentiviral IN proteins from proteasomal degradation, a property that is dependent on IBD-binding but not on chromatin binding or on the cellular location of the IBD-IN interaction [20]. Using the previously validated IN stability rescue assay [20], we found that artificially chromatin-tethered chimeras also protected IN protein from proteasomal degradation (Figure 4B). GFP-H1.1 or GFP-H1.5 had no effect, but each of the chimeric proteins was able to protect IN similarly to LEDGF/p75 (Figure 4B). This protection effect can also be discerned by comparing Figure 3B with Figure 3C–E. GFP-H1.1 and GFP-H1.5 do not rescue IN stability, and the latter is often not visualized well without providing additional IN by transient transfection (bottom row of panel 3B).

Taken together, these experiments demonstrate bona fide IN-to-chromatin tethers that link to IN via an IBD that is functionally normal (i.e. it both binds IN and protects it from degradation in cells), bind chromatin throughout the cell cycle, yet also produce different local intra-chromatin distributions that can even be distinguished by their confocal microscopic patterns.

### Rescue of HIV-1 infection by chimeric proteins

**Figure 5:**
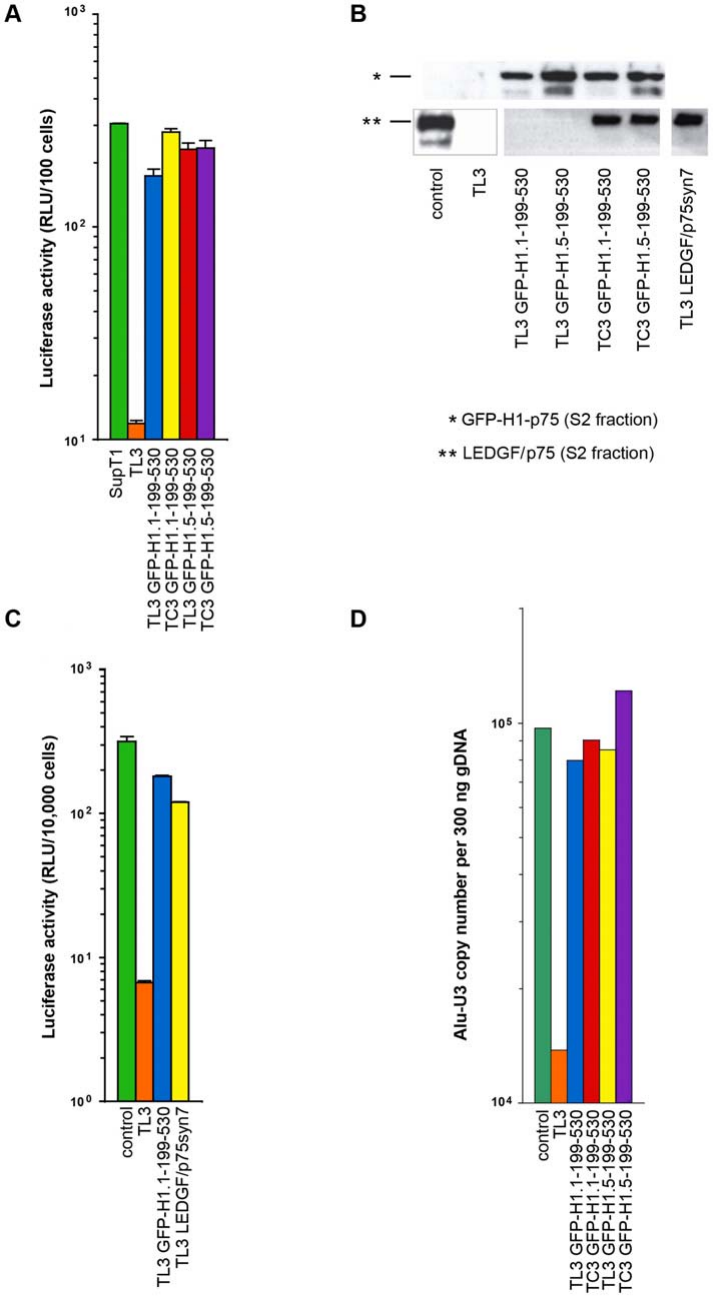
Rescue of HIV-1 infection. (A) SupT1 based cell lines stably expressing H1 chimeras were challenged with VSV-G pseudotyped HIVluc. (B) Cellular fractionation of stable cell lines confirms expression of the H1 chimeras in the S2 fraction and that there is no detectable LEDGF/p75 in the TL3-derived cell lines. (C) Rescue is comparable to that achieved by LEDGF/p75 re-expression (p75syn7). Error bars reflect duplicate measurements in each experiment. (D) Integration assessed by *Alu* PCR.

We next tested effects of chimeric proteins on HIV-1 replication in LEDGF/p75-depleted CD4+ human T cell lines. Table S1 summarizes cell line characteristics. The magnitude of the HIV-1 block in these cells correlates with establishing that the Triton X 100-resistant chromatin fraction is stripped of endogenous LEDGF/p75 [7]. Viral blocks map to integration and are rescued by LEDGF/p75 re-expression but not by LEDGF/p75 deletion mutants lacking either chromatin binding or IBD function [7]. Here, chimeric proteins were expressed in TL3 cells and a paired control shRNA line (TC3 cells) as previously described [7]. The H1-based fusion proteins GFP–H1.1–199-530 and GFP–H1.5–199-530 both rescued single round infectivity as assessed 5 days after infection with HIV-1 luciferase reporter virus (Figure 5A).Time course analysis indicated similar degrees of rescue whether luciferase was analyzed at 24 hours (Figure S2A), or at two months (Figure S2B). Chromatin fractionation confirmed persistent S2 fraction-negativity for endogenous LEDGF/p75 and that the introduced GFP-H1.1-199-530 and GFP-H1.5-199-530 proteins segregated with the S2 fraction (Figure 5B, both S1 and S2 fractions are shown in Figure S2C). Moreover, H1 chimeras had rescuing activity equivalent to LEDGF/p75 itself (Figure 5C) and *Alu*-PCR integration assays showed integration rescue (Figure 5D).

**Figure 6:**
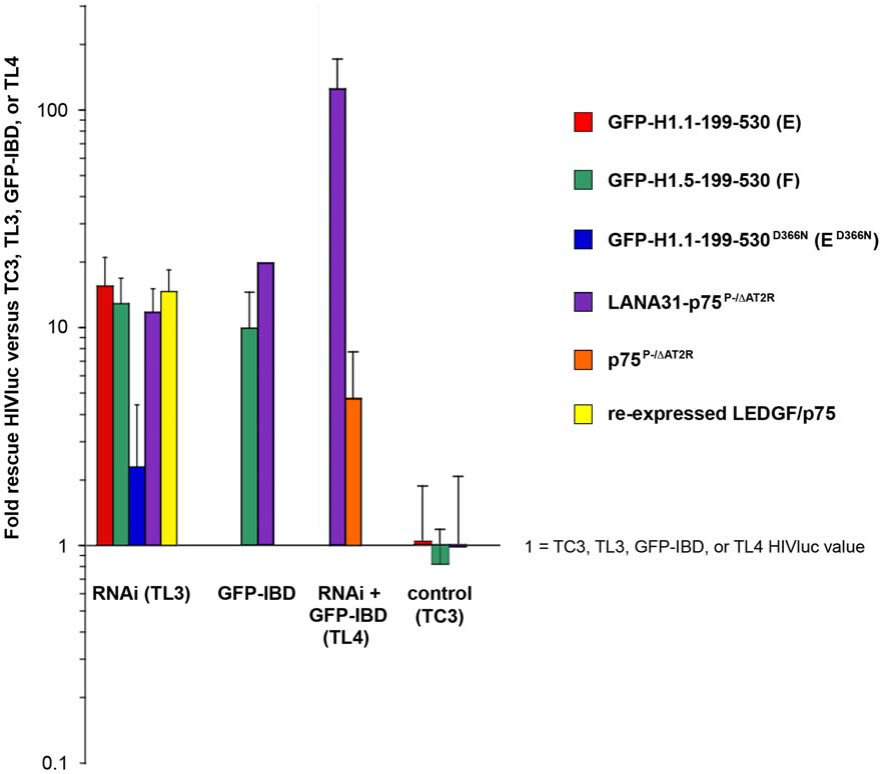
Chimeric proteins rescue infection. Results summarizing multiple experiments are shown as *fold rescue* over baseline of HIV-1luc with the respective chimeras in TL3, GFP-IBD or TL4 cell lines, with the un-rescued cell line values set at 1.0. Experiments were repeated 2–6 times and means+/−S.D. are shown. In TL3 cell-derived lines, some rescue was seen with D366N mutant H1 chimeras, consistent with its markedly impaired but still detectable IN interaction (Figure 4A). LANA31-p75^P-/ΔAT2R^ had similar activity compared to the histone chimeras. TL4 cells express the GFP-IBD dominant interfering protein as well as RNAi against endogenous LEDGF/p75. LANA31-p75^P-/ΔAT2R^ rescued the infectivity defect >two logs in these cells, in contrast to the mutant p75^P-/ΔAT2R^. The fourth column group shows effects of introductions into control TC3 cells, indicating that there is no effect on the viral life cycle when these proteins are introduced without either LEDGF/p75-specific antiviral maneuver (knockdown or dominant interfering protein).

We next tested the LANA31-p75^P-/ΔAT2R^ chimera for its rescuing function and introduced into the analyses the effect of all of the chimeras on not just LEDGF/p75 depletion but also on IBD-mediated dominant interference. Over-expression of the IBD inhibits HIV-1 integration [7],[8],[11]. In such experiments, the IBD has been expressed as a GFP-IBD fusion protein because it is unstable alone (data not shown). Combining stringent lentiviral vector-mediated RNAi knockdown of LEDGF/p75 with GFP-IBD in stable human T cell lines strikingly diminishes HIV-1 infectivity, by over three logs, a high dynamic range that fosters stringent testing of rescue capability (Figure S3). Accordingly, chimeric and control proteins were compared for ability to rescue HIV-1 infectivity defects produced by RNAi alone, GFP-IBD alone, and RNAi plus GFP-IBD in combination. Results are collected and compared as *fold-rescue* data in Figure 6 LANA31-p75^P-/ΔAT2R^ substantially rescued viral infection in the presence of RNAi and competed with the GFP-IBD in GFP-IBD cells, similarly to the H1 chimeras. LANA31-p75^P-/ΔAT2R^ effected a two log rescue in TL4 cells. In contrast, rescue by the parental protein (p75^P-/ΔAT2R^) was minimal. The H1 and LANA chimeras were confirmed to segregate to the chromatin bound S2 fractions of these stable cell lines, while GFP-IBD was in S1 (Figure S4A and B). The ability of H1 and LANA31 chimeras to reverse GFP-IBD dominant interference suggested that it is the location of the IBD that determines its effect, a point we address further in the next Results section. Note that a small yet consistently observed rescuing effect could be detected with a chromatin-tethered D366N mutant (e.g., the effect of GFP-H1.1-199-530^D366N^ in TL3 cells, Figure 6). This effect is consistent with the minimal yet detectable residual interaction between IN and the D366N mutant chimeric protein seen in co-immunoprecipitation experiments (Figure 4A). Similarly, the slight rescue capacity of p75^P-/ΔAT2R^, though consistently observed, was superseded 50-fold by the LANA31 version of this protein (Figure 6). The residual p75^P-/ΔAT2R^ activity may reflect slight chromatin binding activity we have not been able to detect biochemically. Note also that these graphs report the aggregate data for specific cell lines. In individual experiments, up to 15- and 158-*fold* rescues were seen for LANA31-p75^P-/ΔAT2R^ in the TL3 and TL4 cell lines respectively.

### LANA31 peptide-mediated reversal of profound antiviral effects of combined LEDGF/p75 RNAi and IBD dominant interference

**Figure 7:**
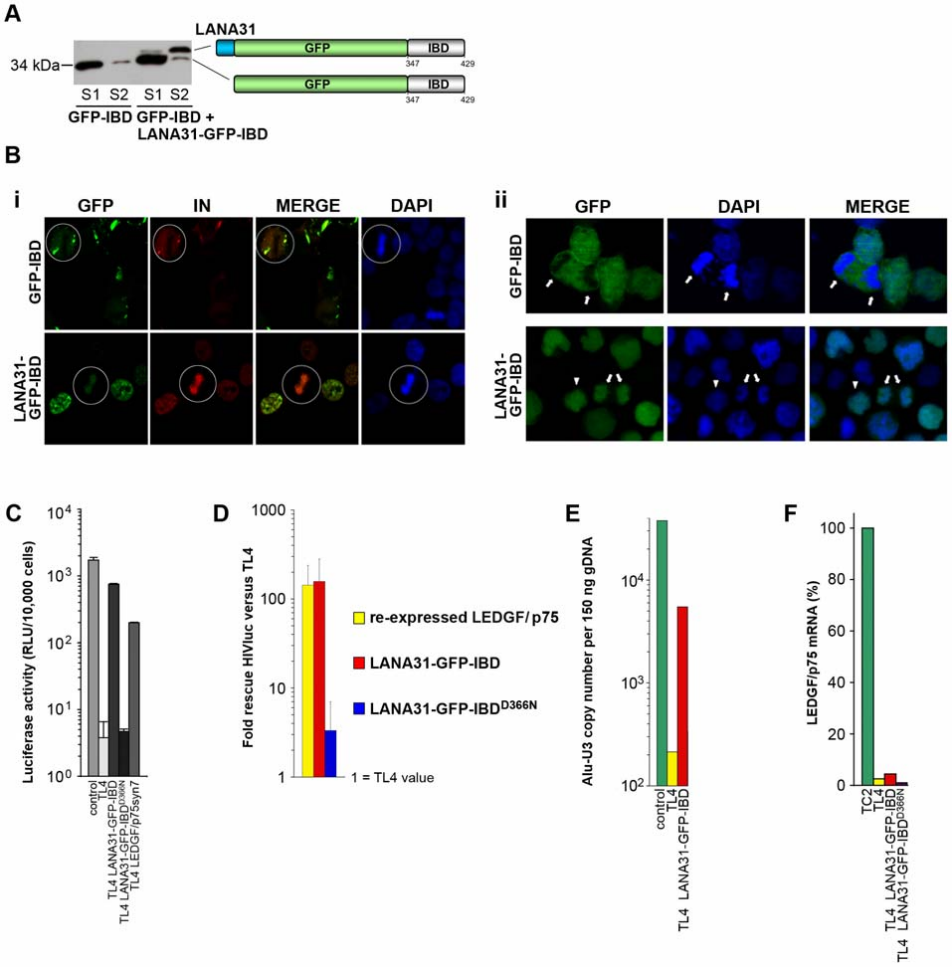
LANA31-GFP-IBD reverses effect of combined LEDGF/p75 depletion and GFP-IBD dominant interference. (A) GFP-IBD and LANA31-GFP-IBD. Amino acid numbers correspond to their position in wild type LEDGF/p75. LANA31-GFP-IBD was expressed by a retroviral vector in TL4 cells. TL4 cells are highly refractory to HIV-1 infection because they simultaneously express GFP-IBD and an endogenous LEDGF/p75-eradicating shRNA [7]. Subcellular fractionation and immunoblotting of resulting stable cell lines with GFP antibody shows GFP-IBD is mainly in S1 while LANA31-GFP-IBD is mainly in S2. The greater levels of GFP-IBD protein compared to the rescuing LANA31-GFP-IBD reflect that expression of the latter was selected by a co-encoded G418 resistance in an MLV-based retroviral vector, which tends to select for a minimal expression level needed to make cells drug stable, while GFP-IBD is encoded from a CMV promoter in a lentiviral vector followed by GFP-enrichment by FACS. (B) Confocal microscopy of LH4 cells transfected with GFP-IBD or LANA31-GFP-IBD are shown in B(i). (See legend to Figure 3 and Materials and Methods for derivation). Both of these proteins interact with HIV-1 IN, but the intracellular locations contrast markedly. GFP-IBD co-localizes with IN but is not chromatin attached, while LANA31-GFP-IBD is nuclear and clearly tethers IN to chromatin. Mitotic cells are circled. B(ii) Imaging of SupT1 cells stably expressing GFP-IBD or LANA31-GFP-IBD confirmed that the LANA31 peptide converts GFP-IBD to a chromatin attached molecule. Mitotic cells are designated with arrows. Note that these cell lines are polyclonal and express variable levels of the GFP fusions. (C) HIV-1 reporter virus challenge. TL4 cells demonstrate a 456-fold decrease in HIV-1 infectivity. LANA31-GFP-IBD effects a 200-fold rescue, functioning comparably to re-expressed LEDGF/p75 and in contrast to the LANA31-GFP-IBD^D366N^. Error bars reflect duplicate measurements in each experiment. Note that as predicted, re-expression of LEDGF/p75 (by transduction of the LEDGF/p75 syn7 cDNA) rescues the cells back to the level of inhibition produced by the RNAi alone. Rescuing proteins were transduced stably with G418-selectable retroviral vectors. (D) Results summarizing multiple experiments (n = 6) comparing LANA31-GFP-IBD, LANA31-GFP-IBD^D366N^ and LEDGF/p75 are shown. (E) *Alu*-PCR integration assay confirming rescue of integration in TL4 cells expressing LANA31-GFP-IBD. (F) LEDGF/p75 mRNA levels in TL4 cell lines, normalized to Cyclophilin A mRNA levels.

The above experiments suggested chromatin attachment *per se*, rather than interaction with a particular ligand, is the critical function mediated by LEDGF/p75. We then asked whether a truly minimal tether consisting of a heterologous chromatin connector linking only the IBD and no other LEDGF/p75 segments to chromatin would support HIV-1 replication. Remarkably, simply fusing the LANA31 peptide to GFP-IBD (Figure 7A) changed the phenotype of this protein from an integration-inhibiting protein to an integration cofactor capable of rescuing over two logs of HIV-1 infectivity (Figure 7C,D). In fact, these minimally tethered IBD constructs were more effective in reversing RNAi or GFP-IBD effects than the more elaborate H1 or p75^P-/ΔAT2R^ constructs (Figures 6 and 7). Corroborating the results, LANA31-GFP-IBD tethered HIV-1 IN to chromatin as revealed by both microscopy and biochemical fractionation while GFP-IBD alone interacted with IN without tethering it (Figure 7A, B). Note also that LANA31-GFP-IBD is *less* abundant in the cells than GFP-IBD (Figure 7A), yet it effectively rescued HIV-1 from the profound antiviral effects of the RNAi+GFP-IBD combination. Figure 7C shows one such experiment, while Figure 7D shows aggregate fold rescue data for all six conducted; we also found that LANA31-GFP-IBD^D366N^ produces a very small rescue effect as shown, but this is dwarfed by the more than two log effect of LANA31-GFP-IBD. *Alu* PCR confirmed rescue at the level of integration specifically (Figure 7E). In addition, 2-LTR circle levels were equivalent (varying less than 2-fold, data not shown), suggesting that nuclear import is unaffected (and assuming that circular forms have equivalent nuclear stability in the presence of GFP-IBD). To exclude the possibility that the two GFP-IBD proteins were interacting with each other with LANA31-GFP-IBD recruiting GFP-IBD to chromatin, we constructed and co-expressed IBD fusions with GFP spectral variants (Figure S5). In addition, immunoblotting of S2 fractions (Figure S6), and real-time quantitative RT-PCR (Figure 7F) on the cell lines expressing the LANA31-GFP-IBD chimeras confirmed no rebound of endogenous LEDGF/p75 expression over the parental TL4 cell lines. These experiments in Figure 7 eliminate all of LEDGF/p75 except for the IBD from cells, with the only variable being the LANA peptide, thus excluding essential roles for any NDE components. They indicate that it is the location of the IBD – in a chromatin-tethered state – that determines the direction of its effect on HIV-1 infection.

### Chimeric proteins support replicating HIV-1 infection

**Figure 8:**
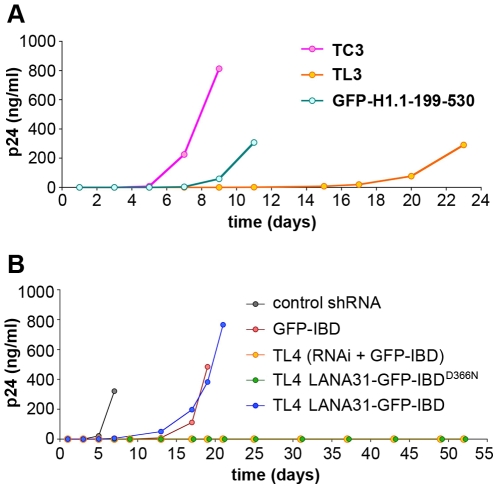
Chimeric LEDGF/p75 proteins support replicating HIV-1 infection. (A) TL3 cells expressing GFP-H1.1-199-530 were challenged with HIV-1 NL4-3 (MOI = 0.01). Supernatants were collected and analyzed for p24. (B) TL4 cell lines expressing LANA31-GFP-IBD or LANA31-GFP-IBD^D366N^ were challenged with HIV-1 NL4-3 (MOI = 0.3). Supernatants were collected and analyzed for p24. GFP-IBD is included for comparison. Cultures were sampled and p24 measured until CPE became overwhelming.

We further examined if these chimeras would function similarly in the context of spreading HIV-1 infection. TL3 cells expressing GFP-H1.1-199-530 were challenged with wild type HIV-1 at MOI of 0.01. As observed previously [7], infection was markedly delayed in TL3 cells, with peak p24 detectable at day 23 compared to control TC3 which peaked at day 9 (Figure 8A). In contrast, more rapid spreading infection occurred in cells expressing GFP-H1.1-199-530 as evidenced by peak p24 detectable at day 11. We also examined the efficacy of LANA31-GFP-IBD in replicating infection as shown in Figure 8B. In control TL4 and TL4 cells with LANA31-GFP-IBD^D366N^ no p24 was detectable at day 55 consistent with profound inhibition of HIV-1 in these cells. Strikingly, LANA31-GFP-IBD rescues infection in TL4 cells to a level similar to that seen in cells that express only GFP-IBD, with peak p24 detected at day 20 in both. Thus, chimeric LEDGF proteins recapitulate wild type LEDGF functions, supporting single round and replicating HIV-1 infections.

### Function of chimeric proteins in LEDGF/p75−/− mouse embryonic fibroblasts

**Figure 9:**
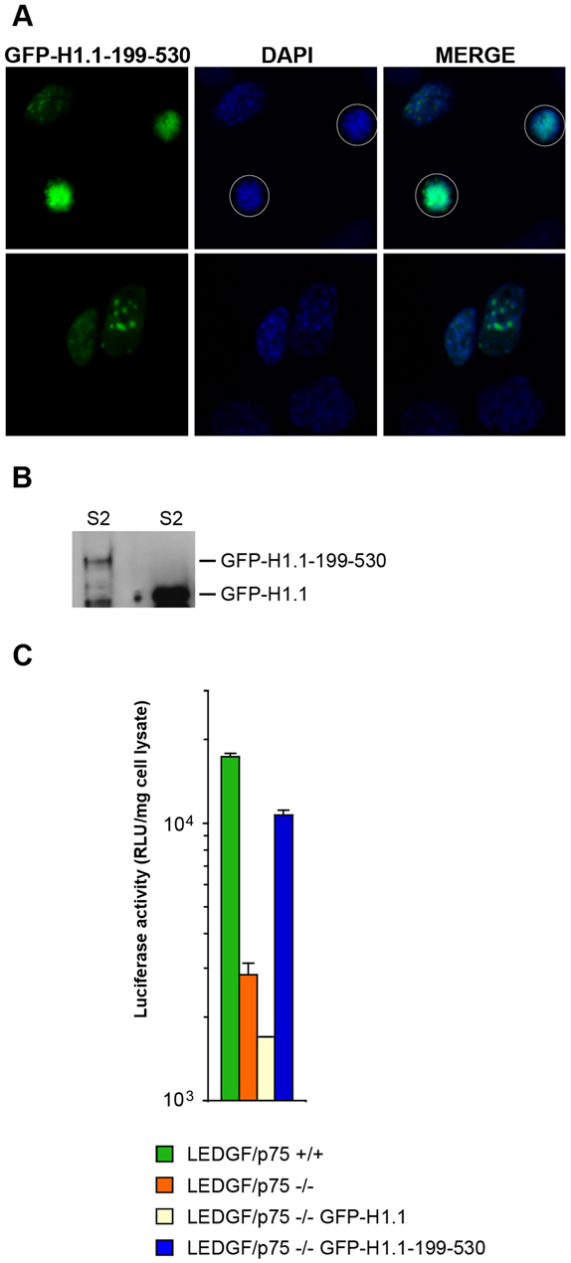
Chimeric LEDGF/75 proteins function in murine LEDGF/p75−/− cells. (A) GFP-H1.1-199-530 (chimera E) protein localization in LEDGF/p75−/− MEFs. Images were obtained 24 hours after transfection. (B) Subcellular fractionation of transfected LEDGF/p75−/− MEFs, western blotting with antiGFP antibody, only S2 fraction shown. GFP-H1.1-199-530 (lane 1) and GFP-H1.1 (lane 2) proteins are indicated. (C) HIV-1 reporter virus challenge. GFP-H1.1-199-530 rescues HIV-1c-luc in LEDGF/p75−/− cells compared to GFP-H1.1 which does not. No full length LEDGF/p75 mRNA transcripts were detectable in the LEDGF/p75−/− MEFs (data not shown).

Two groups have generated LEDGF/p75 knockout mice [12],[45]. HIV-1 integration is impaired approximately 5–10 fold in LEDGF/p75−/− mouse embryonic fibroblasts (MEFs), and LEDGF/p75 depletion alters lentiviral integration profiles to a similar extent in mouse and human cells [6],[12],[13]. Murine LEDGF/p75 thus appears to function similarly to human LEDGF/p75, in directing integration towards active genes. Here we examined if the behavior of the chimeric proteins would be consistent between human T cells and MEFs. Heterochromatin in mouse cells exists as large pericentric blocks that stain intensely with DAPI [46]. GFP-H1.1-199-530, although it remains attached to chromatin through mitosis (Figure 9A), did not overlap tightly with DAPI in MEFs, consistent with the known preferential binding of H1.1 to euchromatin in mouse cells [33]. −/− MEFs were transfected with GFP-H1.1 or GFP-H1.1-199-530, and challenged with HIV-1 reporter virus. Subcellular fractionation and western blotting confirmed the majority of the transfected proteins to be chromatin bound (Figure 9B, S2 fractions of transfected MEFs). HIV-1c-luc expression was approximately 6 fold less in −/− MEFs compared to +/+ MEFs (Figure 9C). GFP-H1.1-199-530 transfection increased detectable luciferase in −/− MEFs approximately 3.7 fold, whereas *less* luciferase activity was detected in −/− MEFs transfected with GFP-H1.1. Thus, the chimera functions similarly in human and mouse LEDGF/p75-deficient cells.

### Properties of LEDGF/p75^PWWP-^

**Figure 10:**
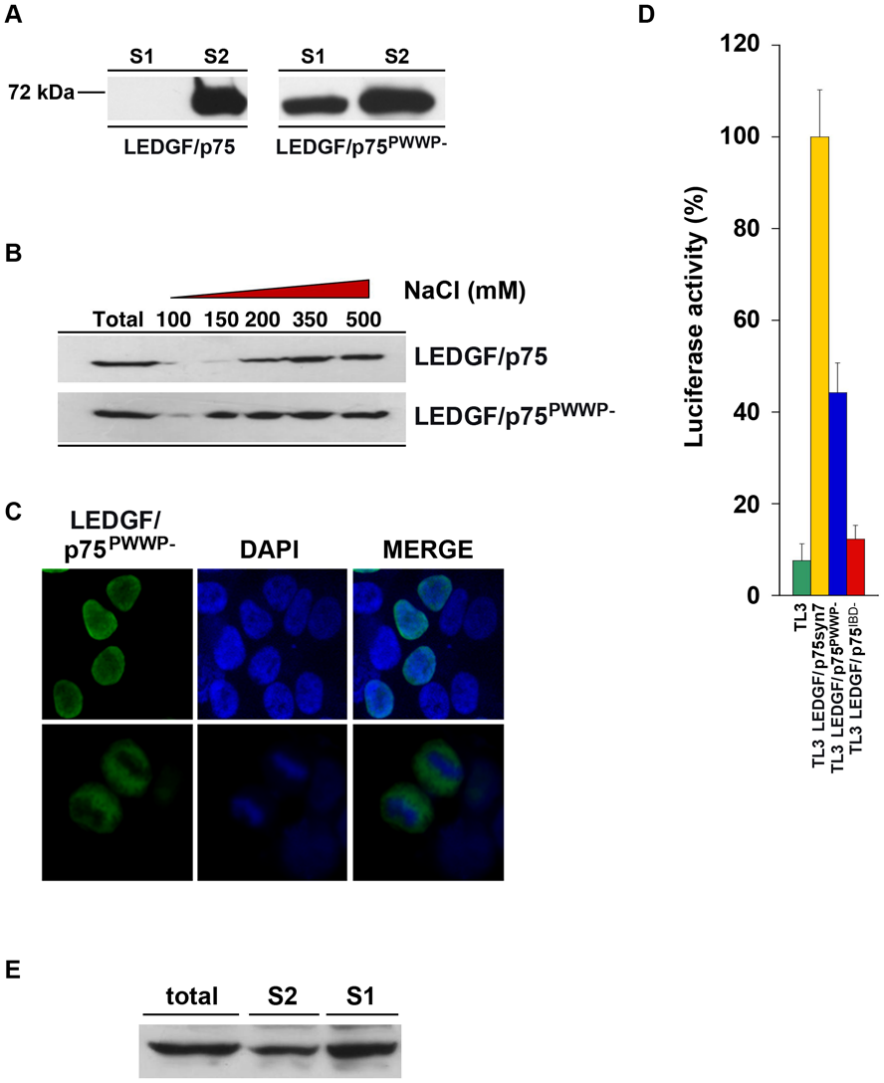
Chromatin association and viral rescue properties of LEDGF/p75^PWWP-^ . (A) LEDGF/p75^PWWP-^ segregates approximately equally to S1 and S2 fractions. L1340 cells were transfected with indicated constructs and chromatin separation protocol performed as described. (B) LEDGF/p75^PWWP-^ is less resistant than LEDGF/p75 to salt extraction from chromatin. Equal numbers of cells were lysed in CSK I buffer containing graded NaCl concentrations or in Laemmli buffer (total fraction). LEDGF/p75 PWWP-deleted LEDGF/p75 is fully extracted from the chromatin in the presence of 150 mM NaCl whereas only trace amounts of LEDGF/p75 wild type are released from chromatin at this ionic strength. (C) Immunofluorescence demonstrates that LEDGF/p75^PWWP-^ is nuclear (top row) but does not bind to mitotic chromatin (bottom row). (D) TL3 cell lines stably expressing LEDGF/p75, LEDGF/p75^PWWP-^, or LEDGF/p75^IBD-^ were challenged with HIV-1luc. Luciferase activity was determined 5 days later and levels are represented as percent of rescue compared to wild type LEDGF/p75. Standard deviations are between fully independent experiments (LEDGF/p75 n = 18, LEDGF/p75^PWWP-^ n = 23, LEDGF/p75^IBD-^ n = 10). (E) TL3-derived cell lines in (D) expressing FLAG epitope-tagged LEDGF/p75^PWWP-^ were subjected to sub-cellular fractionation as described and immunoblotted with anti-FLAG.

Finally, our experiments support a prediction that LEDGF/p75 cofactor activity does not require interaction of its PWWP domain with particular chromatin ligands. To test this in a non-chimeric setting, we employed LEDGF/p75^PWWP-^, a PWWP domain-deletion mutant (Figure 10). This mutant specifically loses mitotic chromatin binding, but it retains strong chromatin binding activity in other cell cycle phases, resulting in its approximately equivalent segregation into S1 and S2, compared to the strict S2 partitioning of LEDGF/p75 [25]. Both properties were re-verified here (Figure 10A,C). Consistent with this, LEDGF/p75^PWWP-^ was less resistant to salt extraction than LEDGF/p75 (Figure 10B). Stable TL3-based cell lines were derived and HIV-1 reporter virus challenges showed LEDGF/p75^PWWP-^ exhibited 44.2+/−6.5% of the rescue activity of wild type LEDGF/p75 (Figure 10D). Thus, the nearly 50% rescue afforded by this PWWP-deleted mutant exactly parallels its chromatin fractionation properties (Figure 10E). Note that further deleting the A/T hook domain from LEDGF/p75^PWWP-^ results in a protein with no S2 fraction association (Figure 2A) and no HIV-1 rescuing function [7].

## Discussion

Our results establish chromatin tethering *per se* as a necessary and sufficient requirement for efficient lentiviral integration and provide further direct evidence that this is the main mechanism of LEDGF/p75 cofactor function in the HIV-1 life cycle. The data work against cofactor models that depend substantially on specific NDE domain interactions with chromatin ligands or on these domains interacting with the IBD region as a functionally integrated protein. Rather, wholesale, structurally diverse substitution is tolerated in the chromatin side of the LEDGF/p75 mechanism. Docking to either nucleosome core proteins or to nucleosome linker DNA is effective. More generally, docking to either DNA or protein molecules in chromatin and docking either internal to the nucleosome or outside of it is sufficient for the HIV-1 co-factor activity of LEDGF/p75. Indeed, the experiments with the minimal construct LANA31-GFP-IBD show that conferring chromatin attachment is sufficient to switch the phenotype of GFP-IBD from integration-blocking to integration-facilitating. In these experiments LANA31-GFP-IBD produced a nearly complete rescue (Figure 7), restoring over two logs of infectivity in these highly HIV-1-resistant cell lines. This result underscores that location of the IBD alone in the cell is the paramount factor.

It has been particularly interesting in this and prior studies that endogenous LEDGF/p75 depletion and IBD over-expression are multiplicative rather than additive, with each producing about 10-30 fold inhibition and the combination reaching over three logs of inhibition. GFP-IBD is dominant interfering rather than dominant negative, because it does not bind LEDGF/p75. That the same protein reverses this combined effect when tethered to chromatin by 31 additional amino acids at the GFP end (and in the presence of the un-tethered form of GFP-IBD) indicates that the dominant interference we are observing is not simply due to decreased catalytic function when GFP-IBD occupies the IN dimer interface or to lack of coupling to NDE functions. Rather, lack of chromatin attachment appears to be crucial.

Retrovirologists have long been intrigued by the important question of how lentiviral pre-integration complex nuclear import is enabled in nondividing cells [47],[48]. However, intensive intellectual focus on this still unsolved question may have diverted attention from a more general problem of retroviral chromatin attachment that has received relatively little investigation yet was recognized to be both important and unsolved a decade ago [2]. The pre-integration complex is intuitively conceived as the active directed participant in the infection process. Insights may be gained by considering the opposite frame of reference, i.e., considering the chromatin fiber as a mobile actor. Indeed, recent nuclear structure/function research provides ample evidence for dynamic chromatin mobility [49]. Without a means to quickly and securely latch onto chromatin after nuclear entry, the pre-integration complex may be at higher risk of attrition. Mammalian genomes record a vast number of retroelement invasions, with 50% of the human genome recognizably derived from retroelements and 8% from endogenous retroviruses [50]. It is likely that the pre-integration complex is vulnerable to any number of evolved intra-nuclear host defense mechanisms. It must not only secure attachment to chromatin, but also avoid sequestration by other nuclear components, whether these are diffusing macromolecules, more fixed elements of a putative nuclear matrix, or specifically evolved nuclear restriction activities. Our data suggest that retroviruses, in common with some large DNA viruses such as EBV, KSHV, and HPV have a need for a chromatin tethering mechanism to allow the viral cDNA to become and remain attached to chromatin as the latter undergoes spatial and/or compositional change. Certain large DNA viruses such as the papilloma viruses and persistent episomal herpesviruses (Epstein-Barr, KSHV) express viral protein tethers that attach their circular unintegrated episomes to chromatin [44], [51]–[55]. From this perspective, retroviruses face the same challenge of establishing and maintaining chromosome association until they achieve chromosomal integration. Similar studies with chimeric fusions have clarified the tethering requirements for EBV [53],[56].

Despite their diversity, the LEDGF/p75 NDE, H1.1, H1.5 and the LANA31 peptide have one functional property in common. Each binds strongly to chromatin in a cell cycle-resilient manner, trafficking in tight association with both condensed mitotic and uncondensed chromatin (Figures 2A,B, 3A,B,C,E, 5B, S1A,B and S2C). LEDGF/p75^PWWP-^, in contrast, loses some rescuing ability. Whether this is due to the specific loss of attachment during the period when chromatin is most mobile (Figure 10C), or to the general loss of avidity detected in the chromatin binding assay (Figure 10A,B,E) is not yet clear. An important aspect to consider is the dramatic transformations in the structural organization of both the cytoplasm and the nucleus that accompanies the cell cycle in higher eukaryotes. Metazoan nuclear envelope reformation after mitosis does not re-enclose mitotically dispersed components. Rather, it occurs by end-telophase coating of condensed chromatin with a lipid membrane bilayer, likely ER-derived, followed by selective nuclear import to regenerate nuclear contents. Thus it is a process of selective re-expansion that at first excludes non-chromatin bound molecules [57]–[59]. Therefore, we hypothesize that a retroviral pre-integration complex without a mechanism to be captured by and stay consistently attached to chromatin throughout the cell cycle may be at risk of attrition during mitosis.

Our results do not discount the importance of the NDE, which merits focused study since the interactions of its elements with particular ligands may in the future represent therapeutic targets. While the basic cofactor role is supported without them, NDE domains surely play more subtle roles in viral replication. One is already known: the lentiviral bias for integration in transcribed gene regions is clearly heavily influenced by LEDGF/p75 [6],[12],[13]. However, this selectivity for active transcription units might in principle also be supported by other nuclear proteins. We speculate that natural selection acting on lentiviral genomes yielded LEDGF/p75 as the mediator of chromatin attachment primarily because it was the best available cellular protein that binds chromatin with high, cell cycle-resilient avidity and to which secure IN binding could also evolve without undue constraints on IN catalysis. It is possible that the other six retroviral genera have evolved alternative tether mechanisms, especially if — as seems plausible given their structural differences and the large surface area involved in the HIV-1 IN dimer-IBD interface — these IN proteins are constrained functionally from evolving dimer interfaces that bind the LEDGF/p75 IBD. For example, it has been suggested that Gag rather than IN serves this role for foamy retroviruses [60].

In the case of HIV-1, the virus has evolved to engage a protein with clear modular tethering functions in its normal cellular roles. This was recently highlighted in several contexts. For example, the LEDGF/p75 IBD interacts with c-Myc interactor JPO2 [61],[62], as well as the menin/MLL histone methyl transferase complex [63], and the pogo transposable element with ZNF domain (pogZ) [64], although the IBD surfaces involved are functionally distinguishable. Both JPO2 and the menin/MLL complex achieve chromatin attachment by interacting with LEDGF/p75, and for menin/MLL oncoproteins this attachment is required for transformation [62],[63]. Fusing the LEDGF/p75 PWWP domain directly to MLL-ENL abrogates the requirement for either LEDGF/p75 or menin in MLL-ENL associated oncogenesis [63]. Thus, the only function of LEDGF/p75 in the trimolecular complex is to tether menin/MLL-ENL to chromatin [63] (reviewed in [65]).

Lentiviruses display an approximately two-fold preference for integrating into active transcription units. This is reduced in LEDGF/p75-depleted cells [12],[13]. Profiling of LEDGF/p75-depleted cells by microarrays has identified modest changes and no specific pattern assignable to particular Gene Ontology subsets suggesting that it does not localize with high specificity [6],[12],[13]. Thus, this protein is likely to associate fairly ubiquitously with chromatin and use of LEDGF/p75 may on average tilt the virus towards transcribed regions favorable for subsequent proviral transcription. In this regard, it may be that some of the differences we observed in luciferase expression and even in integration in cells with introduced chimeras reflect location effects in which the particular chromatin structure (e.g., heterochromatic) may be less hospitable to either integration itself or to transcription of the integrated provirus. Using ligands that target ubiquitous chromatin elements (inter-nucleosome linker DNA segments, core histones), we may be dispersing integration, and subsequent expression levels of the provirus may reflect expression from less transcriptionally favorable integration sites. Answering this question definitively will require the results of work in progress on full-scale comparisons of genome wide integration site distributions with each of these alternative tethers. In addition, more local effects with these artificial tethers having contrasting topologies of nucleosome engagement may be revealing. For example, Wang et al. have shown that HIV-1 integration is favored on the outward facing DNA major grooves of cell nucleosomes [66]. In addition, substituting these alternative tethers with more focused ones could offer opportunities for lentiviral vector targeting. An *in vitro* precedent of concentrating integration at phage lambda repressor sites using a chimera with the IBD or LEDGF/p75 fused to the repressor binding domain has been established [67]. The present results also suggest that the ability of a module, e.g., a designed zinc finger protein, to bind with substantial affinity may impact targeting success.

## Materials and Methods

### Construction of H1.1-LEDGF/p75 and H1.5-LEDGF/p75 chimeras

All LEDGF/p75-chimeric expression constructs used in this work were derived from *p75syn7*, which has 7 synonymous RNAi-blocking nucleotide changes [7]. Well-characterized GFP fusion proteins (GFP-H1.1 and GFP-H1.5) kindly provided by M. Hendzel [33],[39], were the basis for these constructions. GFP refers to the fluorescence-enhanced (eGFP) version. The C-terminal 199–530 amino acids of an shRNA-resistant LEDGF/p75 cDNA, *p75syn7* [19], were amplified with primers 5-NheL199 (AAG CTA GCG TCG ACA TGG TAA AAC AGC CCT GTC CTT) and link-Age-3 (TTA CCG GTT TGC TGC CGC CGC CGC CGG AAT CTA GTG TAG AAT CCT TCA GAG) and ligated upstream of GFP-H1.1 and GFP-H1.5 using Nhe I and Age I to generate constructs A and B. Constructs C and D were constructed similarly using 5 BspESal199 (AAT CCG GAG TCG ACA TGG TAA AAC AGC CCT GTC CTT C) and link-BglII-3 (TTA GAT CTG CTG CCG CCG CCG CCG GAA TCT AGT GTA GAA TCC TTC AGA G) ligated inframe between eGFP and H1.1 or H1.5 using BspEI and BglII. For constructs E and F, the stop codon of H1.1 and H1.5 was first removed using primers 1.5 upstrPST (GCC CAA AGC CAA GAA GGC AG), 1.5noTAGlkBm (TTG GAT CCG CCG CCG CCG GAC TTC TTT TTG GCA GCC GCC TTC),1.1-upstNot (GTG GTG TGT CGT TGG CAG CTC), and 1.1noTAGLkBm (TTG GAT CCG CCG CCG CCG GAC TTT TTC TTG GGT GCC GCT TTC), followed by PCR amplification of LEDGF/p75 199–530 using Bam199 (AAG GAT CCA TGG TAA AAC AGC CCT GTC CTT C) and Xba530 (TTT CTA GAC TAG TTA TCT AGT GTA GAA TCC TTC). For retroviral vector expression, constructs E and F were digested with AgeI, blunted with Klenow polymerase, digested with XhoI and ligated into the Bam-Sal backbone of JZ308 [68], generating JZE and JZF. D366N mutants were generated using overlap extension PCR. All constructs were confirmed by restriction digests followed by DNA sequencing.

### LANA31-LEDGF/p75 chimera

Synthetic oligonucleotides were used to fuse the 31 N-terminal amino acids of KSHV LANA MAPPGMRLRSGRSTGAPLTRGSCRKRNRSPE to the N-terminus of p75^P-/ΔAT2R^, generating LANA31-p75^P-/ΔAT2R^. The functionally critical 23 amino acids mapped by Barbera et al. [29] are underlined. p75^P-/ΔAT2R^ is a LEDGF/p75 chromatin binding domain ensemble mutant in which (i) the 93 N-terminal amino acids of the PWWP domain were deleted and (ii) each of the two A/T hooks was disabled by glycine substitution of a critical arginine residue [69]. p75^P-/ΔAT2R^ does not bind chromatin in immunofluorescence assays that track mitotic chromatin, and displays minimal chromatin binding in the more stringent chromatin binding assay described below. Thus, the protein differs from the H1 fusions in having amino acids 94–198 of LEDGF/p75, except for the Arg to Gly changes at residues 182 and 196, which produce in each A/T hook the disabling RGGP instead of RGRP. The chimeric protein was expressed from a retroviral vector JZ-LANA31-p75^P-/ΔAT2R^, which was constructed as follows. The LANA31 coding sequence was synthesized as forward and reverse oligonucleotides flanked by Bam HI sites (forward-ATATATATATATGGATCCTCGAGATGGCGCCCCCGGGAATGCGCCTGAGGTCGGGACGGAGCACCGGCGCGCCCTTAACGAGAGGAAGTTGTAGGAAACGAAACAGGTCTCCGGAAGGATCCATAT), (reverse-ATATGGATCCTTCCGGAGACCTGTTTCGTTTCCTACAACTTCCTCTCGTTAAGGGCGCGCCGGTGCTCCGTCCCGACCTCAGGCGCATTCCCGGGGGCGCCATCTCGAGGATCCATATATATATATAT). 100 pmol of each strand was annealed in STE buffer (10 mM Tris pH 8, 1 mM EDTA and 50 mM NaCl) by heating to 95°C for 3 minutes and cooling to room temperature. Annealed oligonucleotides were digested with Bam HI inserted into the Bam HI site of JZ-p75^P-/ΔAT2R^. The LANA31-GFP-IBD and LANA31-GFP-IBD^D366N^ were generated by PCR from previously described plasmids [7], using primers 5′BamGFPIBD (ATATGGATCCGTGGTGAGCAAGGGC) and 3′Salp75IBDs (ATATGTCGACCTATCCTTCACCAACCAA). LEDGF/p75^PWWP-^ has been described previously [25].

### LEDGF/p75-targeted RNAi

Studies were conducted in T cell lines rendered endogenous LEDGF/p75-negative, with depletion effective enough to remove detectable protein from the S2 chromatin fraction (see the next section and Table S1 for baseline characteristics of lines used). RNAi was performed with intensified lentiviral vector-based RNAi (ilvRNAi) as described in [7] and reviewed in [70]. One adherent cell line (L cells, described below) was derived alternatively, by stable plasmid-based RNAi, which is fully adequate to report over-expressed HIV-1 IN protein phenotypes. Real time quantitative RT-PCR for LEDGF/p75 mRNA and Cyclophilin A was performed as described [7].

### Cell lines

Human T cell lines were maintained in RPMI with 10% FBS and 293 T cells were maintained in DMEM with 10% FBS, both with penicillin, streptomycin and L-glutamine. Main characteristics of T cell lines used in this work are summarized in Table S1. The previously described **TL3** (active LEDGF/p75-targeted shRNA) and **TC3** (control shRNA) lines are derived from SupT1 cells by ilvRNAi [7]. TC3 and TL3 were established simultaneously from the same parental population, using equivalent MOI transduction with lentiviral vectors that differed only in the 19 nt of the shRNA, followed by equivalent sorting for the co-encoded mCherry marker. Other pertinent phenotypic properties (growth rates, cell morphology, cluster size, CD4 and CXCR4 surface expression, etc.) are indistinguishable [7]. **TL2** and **TC2** are equivalent lines with GFP rather than mCherry co-encoded by the lentiviral vector. **GFP-IBD** cells stably express GFP fused to the IBD (amino acids 347–429) [7]. **TL4** cells are a SupT1 cell line derived by an ilvRNAi vector that expresses the LEDGF/p75-targeting shRNA as well as the dominant-interfering GFP-IBD protein [7]. Whereas TL2 or TL3 cells displayed a 10–30 fold reduction in single round HIV-1 reporter virus susceptibility in HIV-1 infectivity, TL4 cells displayed a 560-fold decrement [7].

Gamma-retroviral (MLV) IN proteins do not interact with LEDGF/p75 [18] and MLV vectors are unimpeded by LEDGF/p75 depletion or dominant interference [7]. MLV vectors were therefore used to stably introduce mutant or chimeric proteins. After transduction, cells were selected and maintained in 600 µg/ml of G418. Introduced proteins that contain the LEDGF/p75 C-terminal region all have 7 synonymous mutations in the shRNA target site.

**L cells** are 293T cells depleted of LEDGF/p75 by stable plasmid-mediated shRNA expression (hygromycin-selected) cytoplasm [18]. **LH4** cells are 293T cells that stably express Myc epitope-tagged HIV-1 IN (puromycin-selected) in the L cell background [19],[20]. LH4 cells are maintained in 3 µg/ml puromycin and 200 µg/ml hygromycin.

### Mouse embryonic fibroblasts

LEDGF/p75+/+ and −/− mouse embryonic fibroblasts (MEF) were obtained from Wendy Bickmore [45]. The lentiviral defect in these cells has been described [13]. The cells used here were harvested 13.5 dpc and immortalized by repeated passaging. MEFs were cultured on gelatin coated plates in DMEM with 15% FBS, 1% NEAA, 7.15 µM beta mercaptoethanol, 1% sodium pyruvate, 1% glutamine and 1% penicillin/streptomycin. LEDGF/p75−/− cells were transfected with GFP-H1.1 or GFP-H1.1-199-530, FACS sorted for GFP expression then either plated for challenge with HIV-1c-luc, or analyzed for protein expression after subcellular fractionation. Infectivity was assessed 72 hours after challenge using BrightGlo (Promega) according to the manufacturer's instructions, and luciferase activity normalized to protein content.

### Vector production

ilvRNAi vectors were produced as described [7],[70]. MLV vectors were produced in 293T cells by calcium phosphate co-transfection of the transfer vector with pHIT60 and pMD.G. Supernatants were collected 48 hours later, filtered (0.45 µM), concentrated over a sucrose gradient and stored at (−) 80°C.

### Virus production, quantification, and titration

Full length HIV-1 NL4-3 viruses were generated in 293T producer cells by calcium phosphate-mediated transfection. Viral particles were quantified in cell supernatants by HIV-1 p24 antigen capture enzyme-linked immunosorbent assay (Zeptometrix, Inc.). Virus titers were determined on GHOST cells.

### Assessment of HIV-1 replication

10^6^ cells were infected with HIV-1 NL4-3 at MOIs of 0.01 or 0.3 in 3 ml RPMI. Cells were washed 4 times after 16 hours. Cells were maintained in 6 ml RPMI. Supernatants for p24 measurement were taken in duplicate periodically and HIV-1 p24 antigen was measured as described above.

### Immunoblotting

Total cell lysates were lysed in RIPA (150 mM NaCl, 0.5% deoxycholate, 0.1% sodium dodecyl sulfate, 1% NP-40, 150 mM Tris-HCl pH 8.0) with added protease inhibitors (complete-Mini, Boehringer), clarified, and protein concentration was determined using the Bradford assay. Fractions and lysates were boiled in Laemmli with ß-mercaptoethanol for 10 minutes, electrophoresed on 10% Tris HCl gels (Biorad) and transferred overnight to Immobilon P membranes (Millipore). Blocked membranes were incubated overnight with primary antibodies as follows: anti-GFP (Clontech, JL8) 1∶5000, anti-myc (9e10, Covance) 1∶500, rabbit anti-myc (Santa Cruz) 1∶500, anti-LEDGF/p75 mAb (BD Biosciences 611714) 1∶500, rabbit anti-LEDGF/p75 (Bethyl Laboratories A300-848A), anti-LEDGF/p75 (Cell Signaling Technologies) 1∶500, or mAb to alpha-tubulin (clone B-5-1-2, Sigma) 1∶8000.

### Chromatin fractionation and salt extraction assays

The fractionation protocol has been characterized extensively [7],[25]. Briefly, cells were lysed for 15 min on ice in cold CSK I buffer (10 mM Pipes, pH 6.8, 100 mM NaCl, 1 mM EDTA, 300 mM sucrose, 1 mM MgCl_2_, 1 mM DTT) supplemented with 0.5% Triton X-100 and protease inhibitors (Roche Complete Mini). Lysates were centrifuged at 500×*g* at 4°C for 3 min. The supernatant (S1 fraction) contains Triton-soluble proteins. The pellet (P1) was resuspended in CSK II buffer (10 mM Pipes, pH 6.8, 50 mM NaCl, 300 mM sucrose, 6 mM MgCl_2_, 1 mM DTT), treated with DNase (1 unit/100 µl) for 30 min, followed by extraction with 250 mM NH_2_SO_4_ for 10 min at 25°C. The DNase- and salt-treated sample was centrifuged at 1,200×*g* for 6 min at 4°C and the supernatant (S2 fraction, containing released chromatin-associated proteins) was collected. The S1 and S2 fractions were analyzed by immunoblotting. P2, which contains non-chromatin bound, Triton-insoluble nuclear proteins such as those comprising the nuclear matrix, and is LEDGF/p75-negative, was not analyzed here. For testing graded NaCl concentrations, 6×10^6^ cells were centrifuged at 1000 g for 6 min at 4°C and pellets were resuspended in 100 µl of CSK I buffer supplemented with protease inhibitors and varying concentrations of NaCl. A total fraction was obtained by re-suspending in 100 µl of Laemmli buffer. After 15 min incubation on ice, the samples were centrifuged at 16,000 g for 2 min at 4°C. Twenty µl of each supernatant was analyzed by immunoblotting.

### Immunoprecipitation

30 µl of Dynal Dynabeads Sheep anti-mouse IgG (product no. 110.31) were blocked in 10% milk in Tris buffered Saline with 0.1% Tween (TBST) by rotating for 1.5 hours at 4°C. 2.8×10^6^ LH4 cells were plated in T 75 flasks, and transfected the next day with 10 µg of the chimeric proteins and 3.5 µg of HIV-1 IN Myc. 48 hours after transfection the cells were scraped off the flasks and lysed for 30 minutes in CSKII buffer [7] with DNase. One third volume of 1 M Ammonium sulfate was added and lysates incubated at RT for an additional 30 minutes, then centrifuged at 1300 g for 6 minutes. 500 µl of the cell lysate was incubated with 3 µg of either a monoclonal GFP antibody (BD Biosciences living colors ref. no 632381) or monoclonal LEDGF/p75 antibody (BD Biosciences cat no. 611714) or isotype control for one hour on ice. This was then mixed by continuous rotation with the pre-blocked beads overnight at 4°C. Beads were washed three times with PBS, eluted in 50 µl of 2× Laemli buffer and boiled for 7 minutes at 95°C before being analysed by western blotting as described above.

### Immunofluorescence and confocal microscopy

Two µg of each plasmid was transfected into cells plated in Labtek II chamber slides (1×10^5^ cells/well). Cells were fixed with fresh 4% formaldehyde in PBS for 10 minutes at 37°C, washed with PBS and permeabilized with ice cold methanol for two minutes at room temperature. Fixed cells were blocked in 10% FCS, 20 mM ammonium chloride and PBS for thirty minutes, incubated with the appropriate primary antibodies for two hours, washed in PBS then incubated for one hour with Alexa 594 or 488 secondary antibodies (Invitrogen, diluted 1∶1000). Cells were washed again with PBS and Prolong Gold mounting solution plus DAPI added. Confocal images were obtained using an LSM 510 device as described [19]. To image T cells, 0.5×10^6^ cells were fixed in 4% formaldehyde as described, washed once in PBS then resuspended in 100 µl of PBS. The cell suspension was placed into a cytofunnel (Shandon Single Cytofunnel Cat. no. 132619) on a slide, (Superfrost Plus, Fisher Brand Cat. no. 22-034-979), clamped into a metal cytospin holder and spun at 600 rpm for 5 minutes. Cells were mounted with Prolong Gold with DAPI and imaged using confocal microscopy.

### HIV-1 reporter virus production, challenge and integration assays

HIVluc is HIV-1 NL4-3 that encodes firefly luciferase in the *nef* ORF; 426 nt of *env* are also deleted as described [7]. HIV-1c-luc has been described previously [7]. VSV-G pseudotyped reporter virus was produced in 293T cells by calcium phosphate transfection and stocks were treated with PfuTurbo DNase at 37°C for 45 minutes. Reverse transcriptase (RT) activity was determined in a TopCount NXT microplate luminescence-scintillation counter (Packard) as described [71]. To gauge preparation quality and virus inputs appropriately, RT activities were compared to a known HIV-1 standard titered on SupT1 cells and luciferase activity per RT unit was determined on SupT1 cells. Challenged cells were harvested 5–7 days after infection except where indicated otherwise, for both luciferase activity (Steady Glo, Promega) and DNA extraction for real-time quantitative PCR assays using the Roche LightCycler [7]. Control cells used were either the TC3 or parental SupT1 cell lines, and all luciferase values were normalized to cell number. *Alu* element-U3 PCR and mitochondrial DNA PCR were performed as described [7].

## Supporting Information

Figure S1 Confocal microscopy of linker histone and LANA31 fusion proteins. (A) H1 fusions and control proteins. See Figure 1 for protein architecture. Proteins were expressed in 293T cells and GFP and DNA (DAPI) were co-imaged. Mitotic cells are highlighted by circling. GFP-199-530 is cytoplasmic and not chromatin bound. GFP-H1.1 and GFP-H1.5 are exclusively nuclear and chromatin bound. The GFP-H1 fusions are also tethered to chromatin throughout the cell cycle. (B) Immunofluorescence microscopy of p75^P-/ΔAT2R^ and LANA31-p75^P-/ΔAT2R^ in L cells. Although nuclear in location by virtue of the retained LEDGF/p75 NLS, p75^P-/ΔAT2R^ does not overlap with DAPI and is not tethered to chromatin. In contrast, LANA31-p75^P-/ΔAT2R^ overlaps with DAPI, and remains tethered to mitotic chromatin throughout the cell cycle. This can even be appreciated in the interphase cells (second panels from left) where p75^P-/ΔAT2R^ is diffusely and homogenously distributed in the nucleus and LANA31-p75^P-/ΔAT2R^ is variegated.
(0.97 MB TIF)

Figure S2 Chimera function analysis at different time points. (A) Luciferase expression in TL3 cells stably expressing GFP-H1.5-199-530 (construct F) analyzed 24 hours after challenge with HIVluc. (B) Luciferase expression in TL3 cell lines stably expressing GFP-H1.1-199-530 (construct E) or GFP-H1.5-199-530 (construct F) analyzed two months after challenge with HIVluc. (C) Subcellular fractions from stable cell lines expressing GFP-H1.1-199-530 or GFP-H1.5-199-530 (analyzed with anti-GFP antibody) or LANA31-p75^P-/ΔAT2R^ (analyzed with anti-LEDGF/p75 antibody).
(0.26 MB TIF)

Figure S3 GFP-IBD interacts with IN and blocks HIV-1 infection. (A) GFP-IBD and IN were expressed by plasmid co-transfection in L cells and imaged by confocal microscopy. Circling highlights a metaphase cell. (B) HIVluc infection of the indicated cell lines. Luciferase activity was measured at 5 days.
(0.46 MB TIF)

Figure S4 Immunoblotting of sub-cellular fractions from stable cell lines. (A) GFP-H1.5-199-530 was stably expressed in cells previously engineered to express GFP-IBD [6]. (B) LANA31-p75^P-/ΔAT2R^ was stably expressed in TL4 cells. The results confirm that the H1 and LANA31 chimeras are confined to the chromatin-bound S2 fraction, while GFP-IBD is found in the non-bound S1 fraction.
(0.19 MB TIF)

Figure S5 Cellular localization of GFP-IBD spectral variants. LANA31-GFP-IBD and mCherry-IBD (or CFP-IBD, data not shown) do not colocalize.
(0.10 MB TIF)

Figure S6 Immunoblotting of sub-cellular fractions of TL4 cells. Western blotting of TL4 cells expressing LANA31-GFP-IBD or LANA31-GFP-IBD^D366N^ confirms that there is no detectable endogenous LEDGF/p75 in the S2 fractions.
(0.08 MB TIF)

Table S1 Baseline characteristics of stable human T cell lines.
(0.03 MB DOC)
