# Supplementary figures and images for: LEDGF/p75 Proteins with Alternative Chromatin Tethers Are Functional HIV-1 Cofactors
Source: PLoS Pathog. 2009 Jul 17;5(7):e1000522. doi: 10.1371/journal.ppat.1000522 (PMC2706977; doi:10.1371/journal.ppat.1000522)

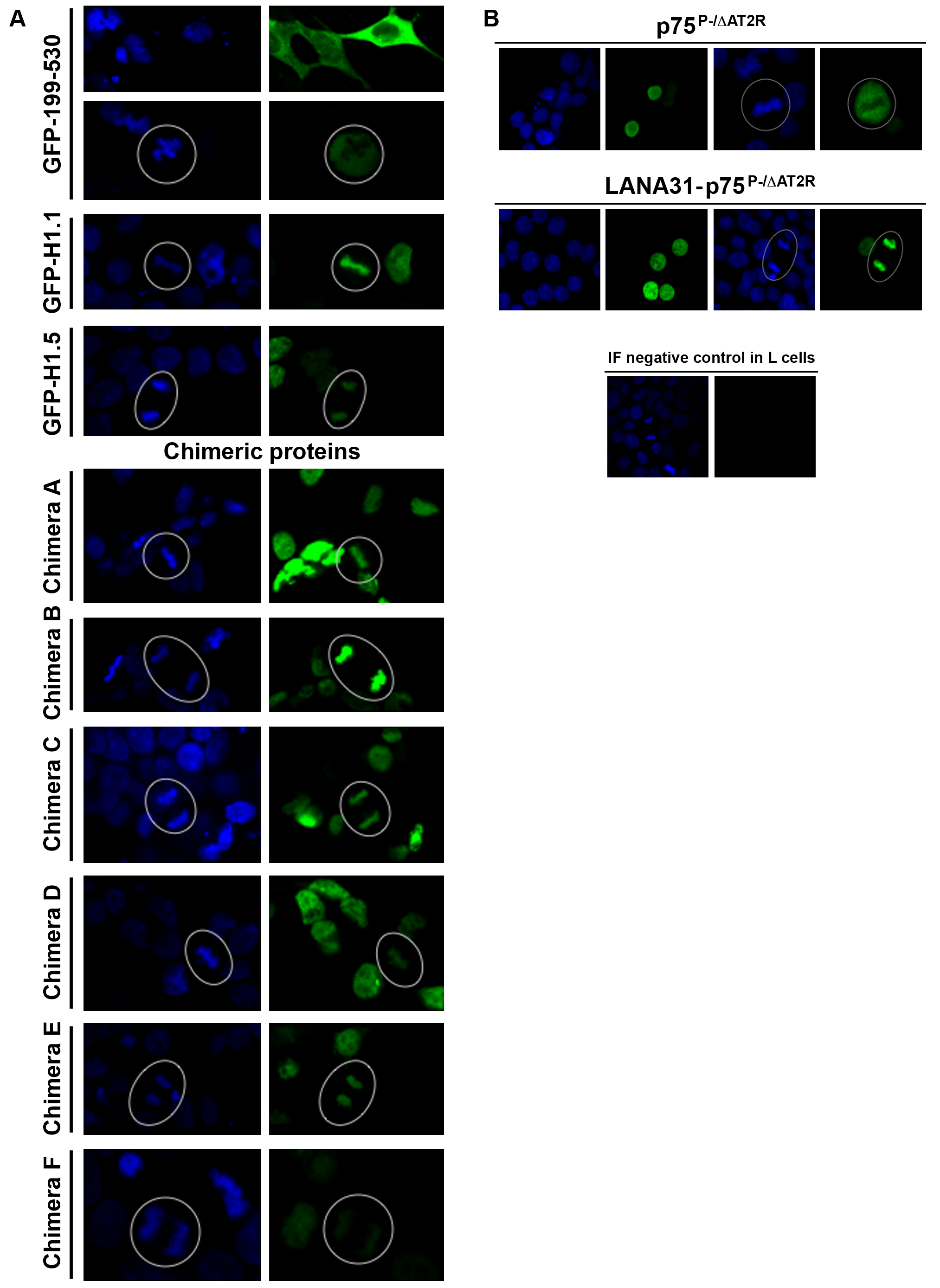

Supplement: Figure S1 — Confocal microscopy of linker histone and LANA31 fusion proteins. (A) H1 fusions and control proteins. See Figure 1 for protein architecture. Proteins were expressed in 293T cells and GFP and DNA (DAPI) were co-imaged. Mitotic cells are highlighted by circling. GFP-199-530 is cytoplasmic and not chromatin bound. GFP-H1.1 and GFP-H1.5 are exclusively nuclear and chromatin bound. The GFP-H1 fusions are also tethered to chromatin throughout the cell cycle. (B) Immunofluorescence microscopy of p75P-/ΔAT2R and LANA31-p75P-/ΔAT2R in L cells. Although nuclear in location by virtue of the retained LEDGF/p75 NLS, p75P-/ΔAT2R does not overlap with DAPI and is not tethered to chromatin. In contrast, LANA31-p75P-/ΔAT2R overlaps with DAPI, and remains tethered to mitotic chromatin throughout the cell cycle. This can even be appreciated in the interphase cells (second panels from left) where p75P-/ΔAT2R is diffusely and homogenously distributed in the nucleus and LANA31-p75P-/ΔAT2R is variegated. (0.97 MB TIF) [file ppat.1000522.s001.tif]

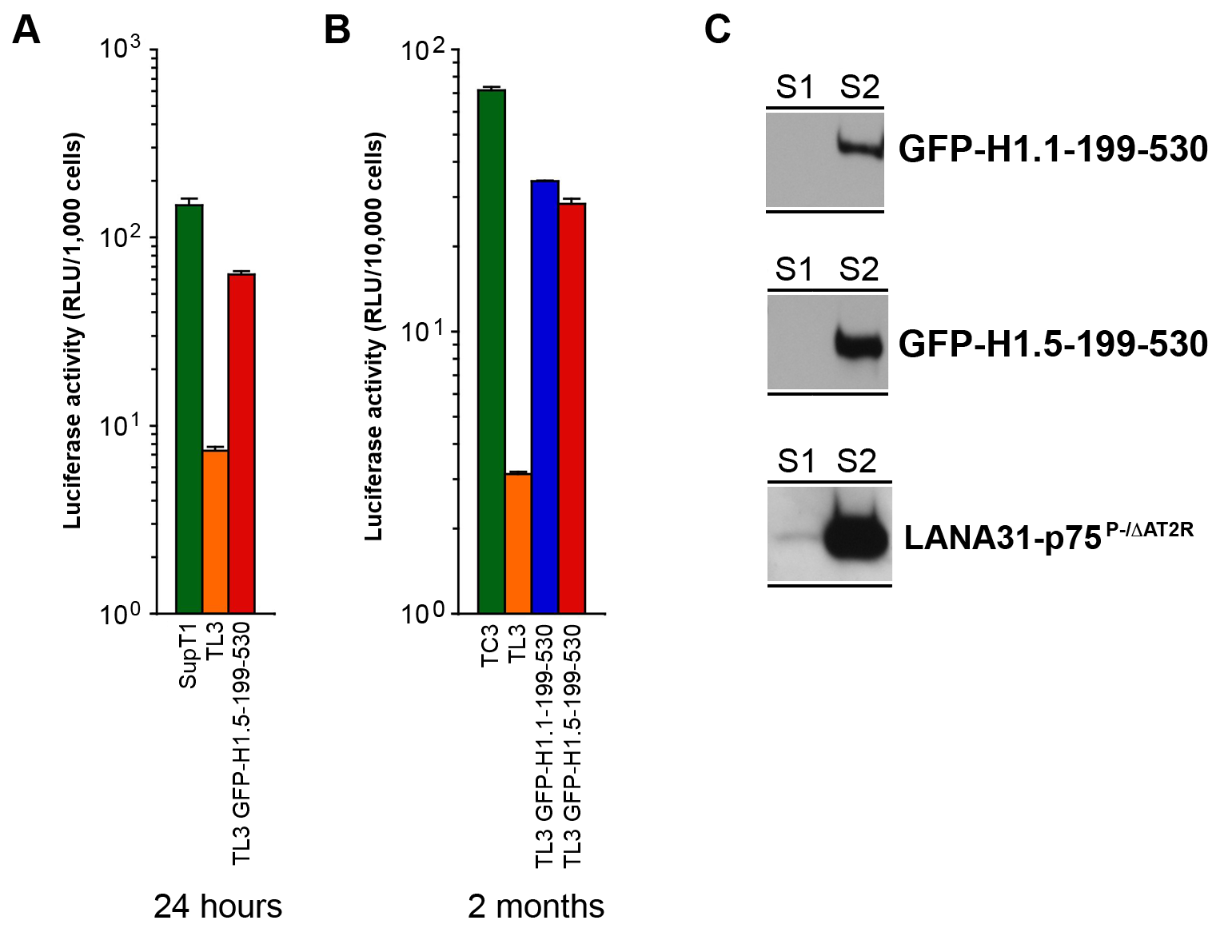

Supplement: Figure S2 — Chimera function analysis at different time points. (A) Luciferase expression in TL3 cells stably expressing GFP-H1.5-199-530 (construct F) analyzed 24 hours after challenge with HIVluc. (B) Luciferase expression in TL3 cell lines stably expressing GFP-H1.1-199-530 (construct E) or GFP-H1.5-199-530 (construct F) analyzed two months after challenge with HIVluc. (C) Subcellular fractions from stable cell lines expressing GFP-H1.1-199-530 or GFP-H1.5-199-530 (analyzed with anti-GFP antibody) or LANA31-p75P-/ΔAT2R (analyzed with anti-LEDGF/p75 antibody). (0.26 MB TIF) [file ppat.1000522.s002.tif]

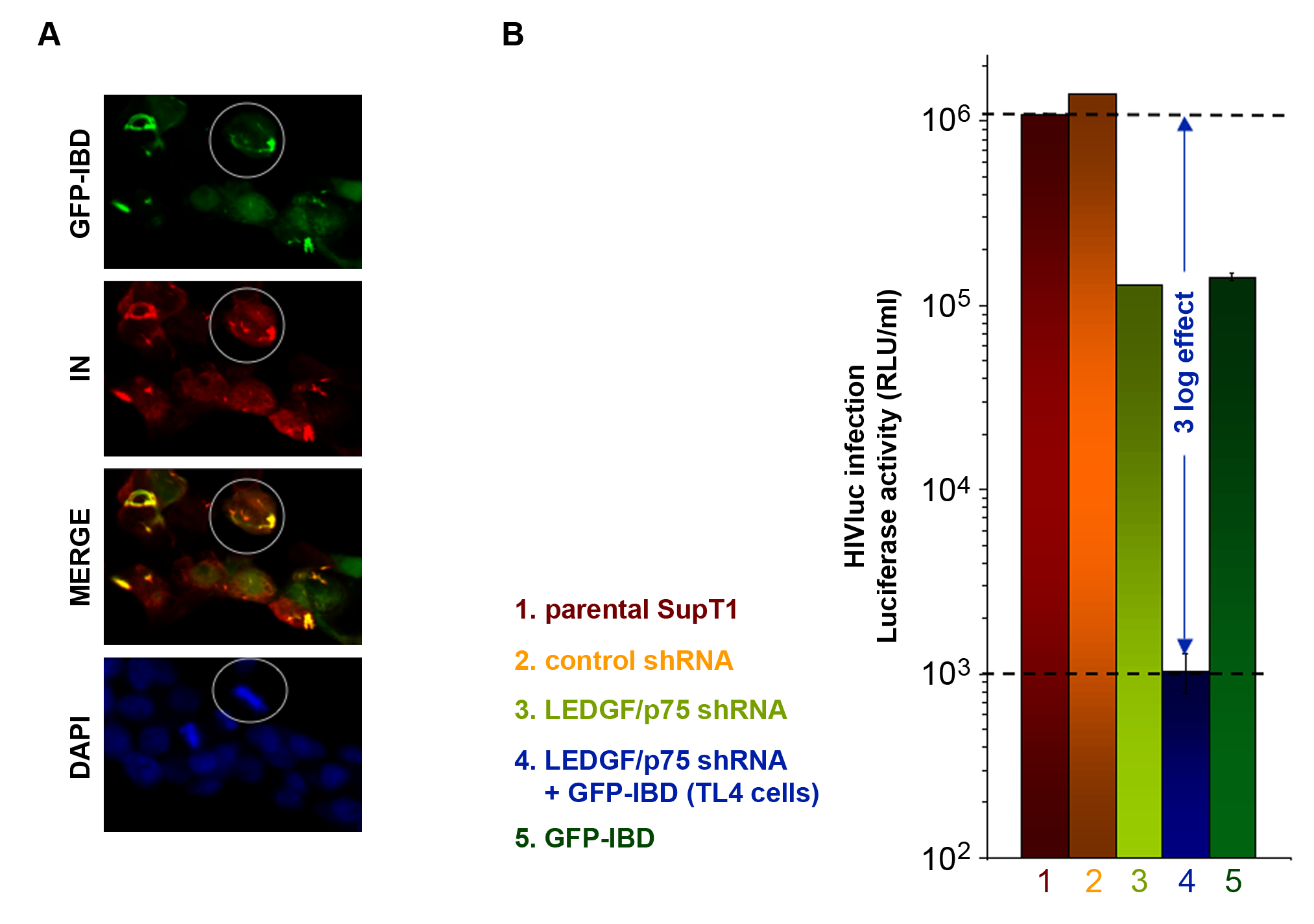

Supplement: Figure S3 — GFP-IBD interacts with IN and blocks HIV-1 infection. (A) GFP-IBD and IN were expressed by plasmid co-transfection in L cells and imaged by confocal microscopy. Circling highlights a metaphase cell. (B) HIVluc infection of the indicated cell lines. Luciferase activity was measured at 5 days. (0.46 MB TIF) [file ppat.1000522.s003.tif]

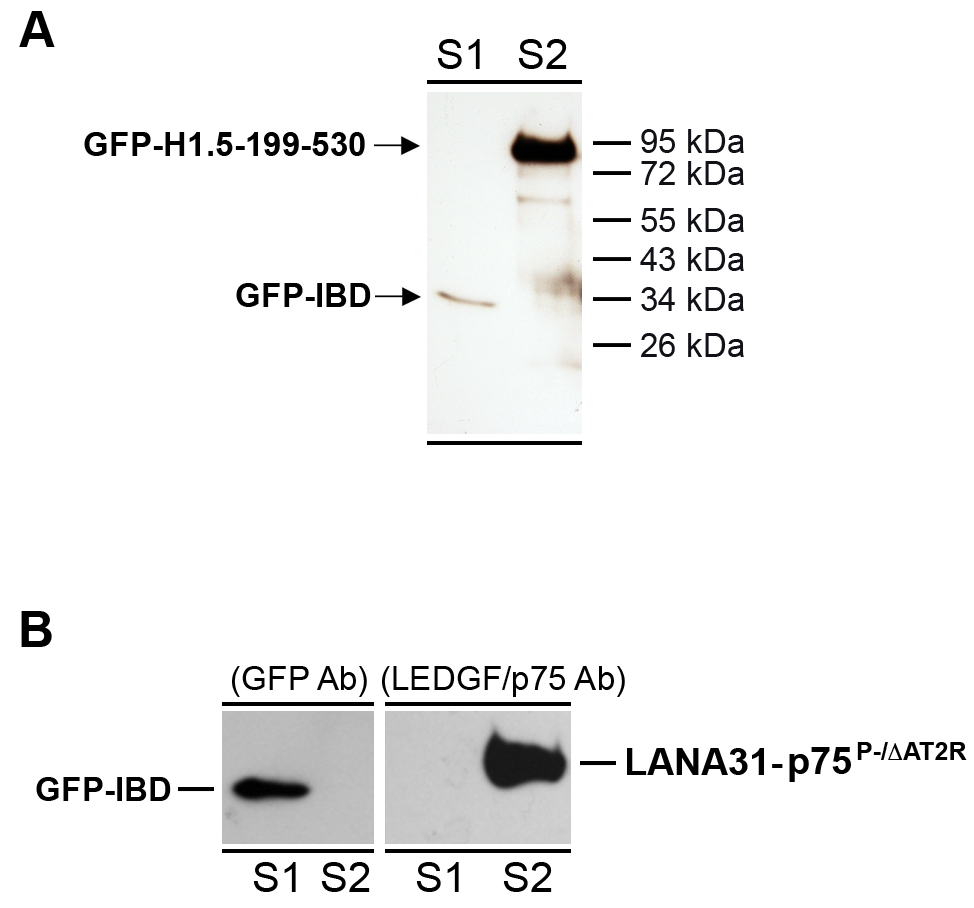

Supplement: Figure S4 — Immunoblotting of sub-cellular fractions from stable cell lines. (A) GFP-H1.5-199-530 was stably expressed in cells previously engineered to express GFP-IBD [6]. (B) LANA31-p75P-/ΔAT2R was stably expressed in TL4 cells. The results confirm that the H1 and LANA31 chimeras are confined to the chromatin-bound S2 fraction, while GFP-IBD is found in the non-bound S1 fraction. (0.19 MB TIF) [file ppat.1000522.s004.tif]

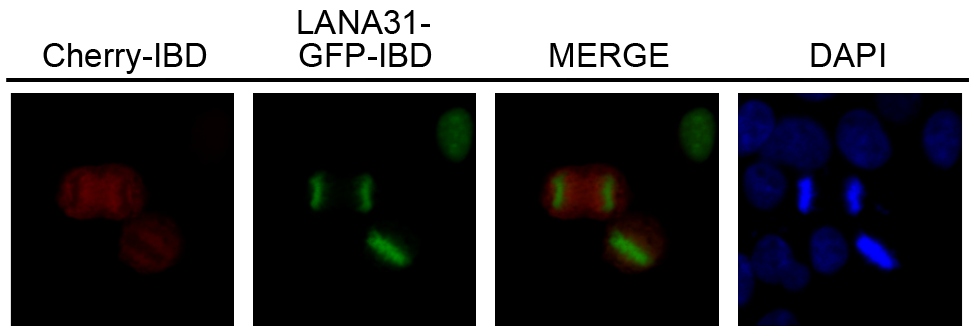

Supplement: Figure S5 — Cellular localization of GFP-IBD spectral variants. LANA31-GFP-IBD and mCherry-IBD (or CFP-IBD, data not shown) do not colocalize. (0.10 MB TIF) [file ppat.1000522.s005.tif]

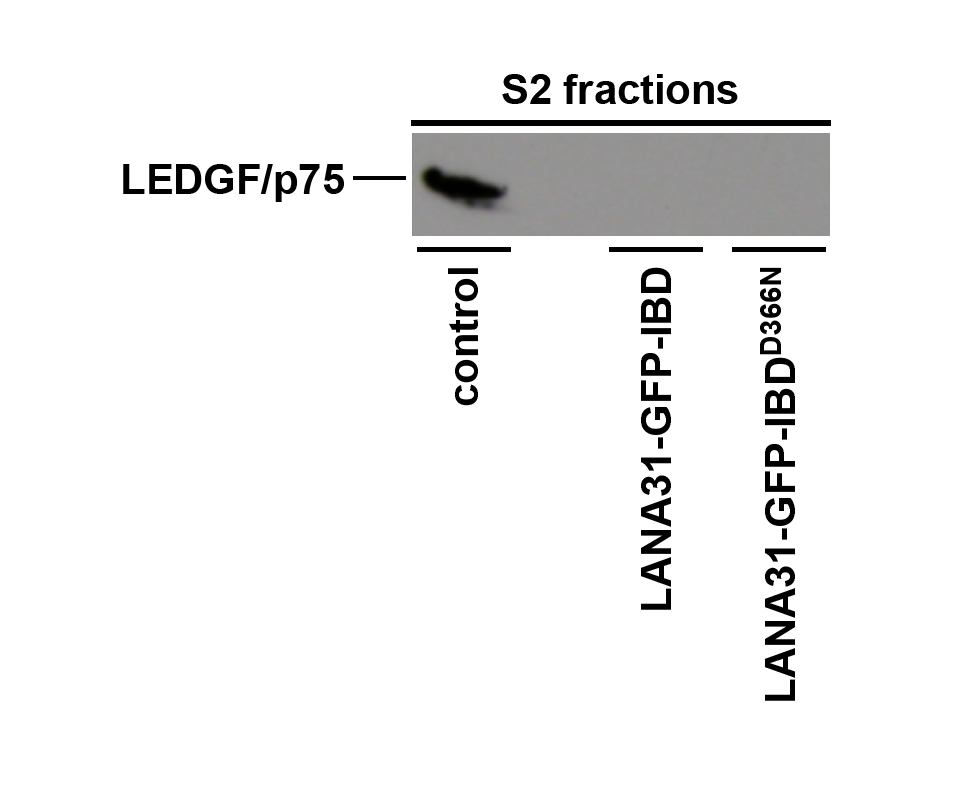

Supplement: Figure S6 — Immunoblotting of sub-cellular fractions of TL4 cells. Western blotting of TL4 cells expressing LANA31-GFP-IBD or LANA31-GFP-IBDD366N confirms that there is no detectable endogenous LEDGF/p75 in the S2 fractions. (0.08 MB TIF) [file ppat.1000522.s006.tif]
