# Supplementary material for: LEDGF/p75 Proteins with Alternative Chromatin Tethers Are Functional HIV-1 Cofactors
Source: PLoS Pathog. 2009 Jul 17;5(7):e1000522. doi: 10.1371/journal.ppat.1000522 (PMC2706977; doi:10.1371/journal.ppat.1000522)
Supplement: Table S1 — Baseline characteristics of stable human T cell lines. (0.03 MB DOC) [file ppat.1000522.s007.doc]

**Table S1. Baseline characteristics of stable human T cell lines**

| **Cell line** | **shRNA** | **Fluorescent Protein** | **Fold change in luciferase activity*** |
| --- | --- | --- | --- |
| SupT1 | — | — | — |
| TC2 | Control | GFP | 0-2 **↑** |
| TL2 | LEDGF/p75 | GFP | 10-30 **↓** |
| TC3 | Control | mCherry | 0-2 **↑** |
| TL3 | LEDGF/p75 | mCherry | 10-30 **↓** |
| GFP-IBD | None | GFP-IBD | 10-50 **↓** |
| GFP-IBD^D366N^ | None | GFP-IBD^D366N^ | 0-3 **↓** |
| TL4 | LEDGF/p75 | GFP-IBD | 400-1600 **↓** |

*Fold-change compared with simultaneously tested parental SupT1 cells. Luciferase was assayed 5 days after infection with VSV-G pseudotyped HIV-1 reporter virus HIVluc. Data are from Llano, et al., *Science* 314, 461-464 as well as subsequent experiments in the present work (e.g., in [6], HIVluc was inhibited 560-fold in TL4 cells; see also Fig. S2). The LEDGF/p75-specific short hairpin RNA produces a knockdown of potency sufficient to remove detectable endogenous LEDGF/p75 from the S2 fraction, i.e., the chromatin-associated protein fraction that resists Triton-X 100 extraction [6]. This strongly bound component is only released by dissolution of chromatin with DNAse and high salt treatment. These S2 fraction-negative (S2FN) human CD4+ T cells display 10- to 30-fold deficits in single round HIV-1 reporter virus susceptibility and substantially delayed HIV-1 replication curves [6].
